# A high-resolution network model for global gene regulation in *Mycobacterium tuberculosis*

**DOI:** 10.1093/nar/gku777

**Published:** 2014-09-17

**Authors:** Eliza J.R. Peterson, David J. Reiss, Serdar Turkarslan, Kyle J. Minch, Tige Rustad, Christopher L. Plaisier, William J.R. Longabaugh, David R. Sherman, Nitin S. Baliga

**Affiliations:** 1 Institute for Systems Biology, 401 Terry Ave N, Seattle, WA 98109, USA; 2 Seattle Biomed Research Institute, 307 Westlake Avenue North, Suite 500, Seattle, WA 98109, USA

## Abstract

The resilience of *Mycobacterium tuberculosis* (MTB) is largely due to its ability to effectively counteract and even take advantage of the hostile environments of a host. In order to accelerate the discovery and characterization of these adaptive mechanisms, we have mined a compendium of 2325 publicly available transcriptome profiles of MTB to decipher a predictive, systems-scale gene regulatory network model. The resulting modular organization of 98% of all MTB genes within this regulatory network was rigorously tested using two independently generated datasets: a genome-wide map of 7248 DNA-binding locations for 143 transcription factors (TFs) and global transcriptional consequences of overexpressing 206 TFs. This analysis has discovered specific TFs that mediate conditional co-regulation of genes within 240 modules across 14 distinct environmental contexts. In addition to recapitulating previously characterized regulons, we discovered 454 novel mechanisms for gene regulation during stress, cholesterol utilization and dormancy. Significantly, 183 of these mechanisms act uniquely under conditions experienced during the infection cycle to regulate diverse functions including 23 genes that are essential to host-pathogen interactions. These and other insights underscore the power of a rational, model-driven approach to unearth novel MTB biology that operates under some but not all phases of infection.

## INTRODUCTION

*Mycobacterium tuberculosis* (MTB) is an extraordinarily successful pathogen that has infected thirty percent of the world's population (http://apps.who.int/iris/bitstream/10665/91355/1/9789241564656_eng.pdf). The success of MTB is tied to the adaptive repertoire of the bacilli in the face of varying and hostile environments within the host. In the course of chronic infection, MTB encounters diverse environmental conditions, including hypoxia, nitric oxide stress and varying nutritional limitations (1). Microbes respond and adapt to such immunological, environmental and nutritional changes through regulatory programs primarily encoded at the transcriptional level. A significant fraction of these regulatory programs are controlled via transcription factors (TFs) that modulate transcriptional activity upon binding to *cis*-regulatory motifs located in intergenic promoters. A detailed model of MTB's transcriptional regulatory network, including the complete set of TFs, co-regulated genes, and regulatory motifs, has significant implications for elucidating novel strategies to eradicate infection by MTB.

Models of transcriptional regulatory networks are typically constructed by integrating large omics datasets with computational algorithms to reproduce and elucidate complex regulatory interactions. Through an iterative process, network models can inform the design of new biological experiments, which yield more powerful models. Bridging the gap between computation and experimentation has remarkable promise, especially in organisms like MTB that are challenging and time-consuming to work with in the laboratory.

Useful information about MTB's transcriptional regulation is available from studies that have focused on regulation during particular stages of pathogenesis such as hypoxia (2), transition to growth arrest (3) or macrophage infection (4). However, the aforementioned models cover at most 50% of the MTB genome. To improve on this, we reconstructed a global transcriptional regulatory network model of MTB that encompasses up to 98% of the genome (3922 genes) and accurately predicts gene expression for new environmental conditions.

In this study, we present a comprehensive transcriptional regulatory network of MTB. Our method generated an environment and gene regulatory influence network (EGRIN) of MTB using a compendium of 2325 publicly available mRNA expression profiles (5). Subsets of putatively co-regulated genes (i.e., biclusters) were identified based on coherent mRNA expression across some environmental conditions and the presence of a common promoter TF binding motif. Accuracy of this model was tested by performing new experiments to overexpress all of MTB's ∼200 TFs, followed by ChIP-Seq (chromosome immunoprecipitation followed by sequencing) and expression profiling. We demonstrate that the EGRIN model accurately predicts regulatory interactions in a network of 240 validated biclusters. We augmented these validated biclusters with gene ontology (GO) terms for enrichment of genes in each bicluster (6) and identified the environmental contexts in which they act by analyzing transcriptional responses across different environmental and nutritional conditions. Following validation, we used the EGRIN model to investigate how cholesterol utilization genes are co-regulated. MTB uses host-derived cholesterol as a carbon source, a facility that is required for MTB persistence in the host (7). We analyzed the regulatory mechanisms responsible for this important carbon shift and uncovered novel interactions for testing in new environmental and genetic perturbation experiments. Similarly, the model has also discovered putative previously uncharacterized regulons that are active across 14 environmental contexts, including hypoxia, carbon monoxide stress, and nitrosative stress.

The MTB expression data (http://www.tbdb.org) (8,9), cMonkey algorithm (http://baliga.systemsbiology.net/drupal/content/cmonkey), MTB EGRIN model and validation data (http://networks.systemsbiology.net/mtb/) are available online. By making our approach and results publicly available, we are encouraging exploration of the model to drive rational experimentation. The EGRIN model is sufficiently predictive to formulate hypotheses of MTB regulatory interactions that respond to various environmental conditions, including those responsible for MTB pathogenicity.

## MATERIALS AND METHODS

The approach used in this study includes both computational and biological methods. Unless otherwise noted, all algorithms developed for this research were implemented in the R programming language (10). Plots were generated using R (10), regulatory network diagrams were generated using BioTapestry (11) and images were prepared using Adobe Illustrator CS5.

### Construction of environmental and gene regulatory influence network

The cMonkey integrated biclustering algorithm was applied to identify subsets of genes that were co-regulated under certain culture conditions (12). The inputs to cMonkey were 2325 transcriptome profiles (Supplemental Data file S1), upstream regions of all genes, and functional association networks, including operon predictions from MicrobesOnline and functional protein interactions from EMBL String databases (13). Briefly, the transcriptome data were microarrays obtained as pcl files from TBDB (www.tbdb.org). Each file was standardized independently as described previously (13) and merged into a single file by gene name. During clustering analysis, cMonkey iteratively prioritizes the grouping of genes with similar expression profiles, supported by additional evidence of co-regulation such as the existence of similar *cis*-regulatory motifs in their promoter regions (detected *de* *novo* using the *MEME* algorithm) (14) and functional associations between genes (functional association network provided by STRING database) (15). cMonkey first creates seed clusters and then optimizes them to create biclusters by adding or removing genes and conditions after calculating coexpression measures, searching for motifs and additional evidences of co-regulation. At each stage it computes the probability of being a member of the bicluster for each gene or condition sampled from the conditional probability distribution. The algorithm allows genes to be members of multiple co-regulated gene groups, a property that is consistent with how biology operates, thereby allowing the discovery of combinatorial regulation of the same genes by multiple environmental factors and/or TFs.

### TF overexpression—ChIP-Seq binding analysis

To systematically map TF binding sites, we performed ChIP-Seq using FLAG-tagged TFs episomally expressed under control of a mycobacterial tetracycline-inducible promoter (2). MTB H37RV cells were cultured in Middlebrook 7H9 with ADC (Difco), 0.05% Tween80 and 50 μg ml^−1^ hygromycin B at 37°C with constant agitation and induced with 100 ng ml^−1^ anhydrotetrachycline (ATc) during mid-log-phase growth. ChIP was performed using a protocol optimized for strains of *Mycobacteria* and related species of *Actinomycetes* and sequencing was performed on an Illumina GAIIx sequencer. Full data files, the algorithm used for peak-calling and analyzed ChIP-Seq targets for each TF are available on the network portal (http://networks.systemsbiology.net/mtb/). Motif discovery and analysis from ChIP-Seq binding targets were carried out using MEME and MAST (14).

### TF overexpression—microarray analysis

MTB H37RV cells were cultured and induced as described above. All experiments were performed under aerobic conditions and growth was monitored by OD600. Total RNA was isolated from TF-induced cultures 18 h after treatment with 100 ng ATc per ml of culture or an equivalent volume of DMSO (in the case of uninduced controls). When interrogating the same culture for ChIP-Seq and transcriptome profiling, cells were divided immediately prior to sample processing. RNA samples were isolated from MTB cells and profiled using custom Nimblegen microarrays. Expression ratios were generated by comparing the induced expression level to a baseline median expression value calculated from all the microarrays where the TF was not induced. Altered gene expression was considered significant if it produced a moderated *t*-test *P*-value <0.01 after Benjamini Hochberg multiple testing correction. Expression data are available on the network portal (http://networks.systemsbiology.net/mtb/) and the gene expression omnibus (GEO) in series GSE59086.

### Bicluster enrichment analysis of TF overexpression regulatory targets

We identified biclusters with significant enrichment of regulatory targets of overexpressed TFs by pairwise overlap and computed hypergeometric enrichment *P*-values. The genes of a bicluster with regulatory binding were identified as having a significant overlap with ChIP-Seq binding targets of a particular TF as well as having a significant overlap with differentially expressed genes of the same TF. The significance of overlap was calculated as the number of genes with regulatory binding from a bicluster compared to randomly sampled gene sets of the same size. In total, 50 000 permutations were performed and the significance of the overlap between a TF's regulatory targets (ChIP-Seq targets that are also differentially expressed) and a bicluster's member genes was calculated based on the resulting permuted *P-*values (Benjamini-Hochberg, BH, multiple hypothesis correction ≤ 0.01). The biclusters enriched in ChIP-Seq binding and the biclusters enriched in diffentially expressed genes in bicluster member genes were also calculated separately by pairwise overlap and computed hypergeometric enrichment *P*-values, with significant biclusters having a BH multiple hypothesis corrected *P*-value ≤ 0.01.

### Functional annotations for validated biclusters

GO annotations for each MTB gene were obtained from UniProt-GOA (16). We used the Bioconductor package topGO (17) to discover significantly enriched GO terms in gene sets of interest. Briefly, topGO tests the enrichment of GO terms with validated targets of each TF using two statistical tests, namely Kolmogorov–Smirnov test and Fisher's exact test.

### Discovering context-dependent regulation by TFs

In total, 1355 transcriptome profiles were categorized into 14 condition sets for this analysis (Supplemental Data file S5). We only used experiments from the compendium set that were published with experimental methods to help inform their categorization into a condition set. Correlation coefficients between the expression of a TF and bicluster member genes were calculated for each of the 14 condition sets. Positive and negative correlations were considered separately, where a positive correlation indicates an activator role and a negative correlation coefficient indicates a repressor role. Median correlation coefficients between a TF and bicluster member genes in a condition set were compared to randomly sampled gene sets of the same size. In total, 10 000 permutations were performed and the significance of the median correlation coefficient between a TF and its bicluster member genes under each condition set was calculated based on the resulting permuted *P-*values (BH multiple hypothesis correction ≤ 0.05). As a final test, we performed a Pearson's pairwise correlation of all TFs and the median expression of the bicluster member genes in each condition set. We identified genes that were significantly correlated with the TF's expression under that condition set based on a correlation coefficient −1.0 ≤ *R* ≥ −0.85 or 0.85 ≤ *R* ≥ 1.0 and a *P*-value < 0.05.

### Bicluster enrichment analysis of cholesterol utilization

For bicluster enrichment analysis, a list of genes essential for *in vitro* growth on cholesterol was collected from Griffin *et al.* (18). The genes represented in this list were compared with the members of each bicluster to find statistically significant enrichment of cholesterol utilization. *P*-values for overrepresentation of cholesterol utilization-specific genes in each bicluster were calculated using the hypergeometric distribution and were corrected for multiple hypothesis testing by the BH method.

### Motif analysis of cholesterol utilization biclusters

Motifs (*e*-value < 1) from the cholesterol utilization biclusters were run against the ‘Prokaryotes–RegTransBase v4′ database using TOMTOM (19). The regulators with significant *P*-values (*P* ≤ 0.01) were searched for sequence similarity against the MTB genome using *blastn* (nucleotide query/nucleotide database) from TBDB (8,9). Finally, the genes with lowest *e*-values (*e* ≤ 0.1) were aligned with the corresponding bicluster motifs using MAST (20). The resulting *P*-values were subjected to a cutoff value of 0.01 to determine regulation.

## RESULTS AND DISCUSSION

**Figure 1.**
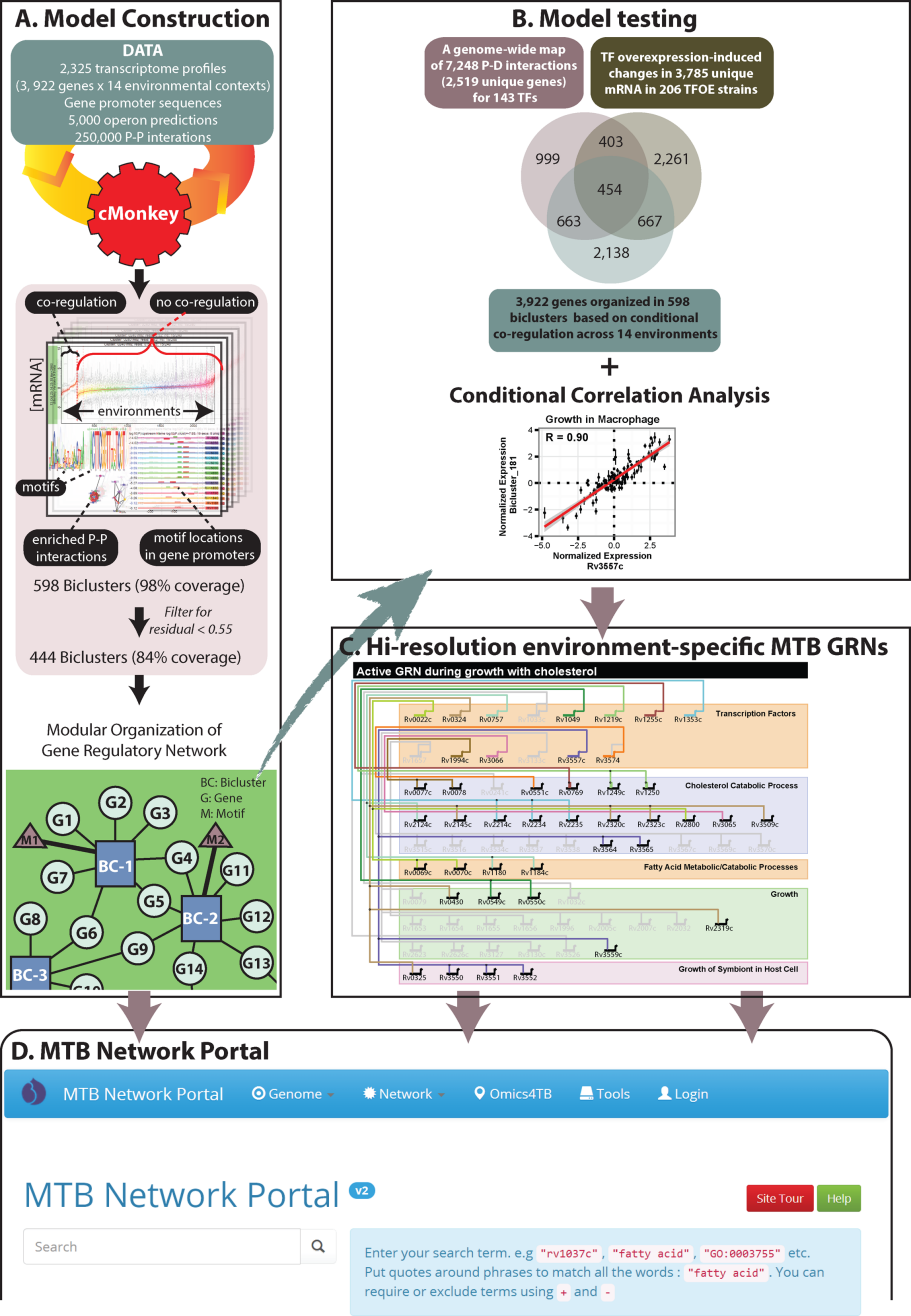
An overview of the approach to model global gene regulation in *Mycobacterium tuberculosis*. The approach consists of four parts. (**A**) The model was constructed using publicly available data. The cMonkey biclustering algorithm identified sets of genes that are co-regulated under a subset of experimental conditions, have a common motif in their promoters and are enriched in protein–protein (P–P) interactions. Biclusters were filtered based on their residuals and the resulting biclusters were organized into a network model of gene regulation. (**B**) The model was tested for accuracy by investigating how often two or more genes within a bicluster were bound by the same TF (P–D interactions) and had mRNA changes upon over-expression of that TF. This identified a set of 454 genes that were co-regulated in varying combinations across biclusters. (**C**) Validated biclusters were investigated for environment-specific regulation patterns of important functions in MTB. (**D**) The MTB Network Portal was developed to allow exploration of the model and enable predictions for experimental investigation. The web-portal is located at http://networks.systemsbiology.net/mtb/.

Our primary goal was to construct a model that could be used to generate hypotheses and guide experimentation to discover and characterize context-specific regulatory mechanisms. In the following sections, we describe the systems approach that was used to investigate genome-wide transcriptional regulation under environmental conditions encountered by MTB during its infection cycle in the human host (Figure 1A–D). In addition to providing details on the global architecture of the resulting network, we discuss insights that were gained from rigorous testing to evaluate model accuracy and utility in making meaningful experimentally testable predictions of context-dependent transcriptional regulation in MTB.

### Reconstruction of a gene regulatory network model of MTB

The MTB EGRIN model was constructed from a compendium of 49 microarray datasets, containing 2327 publicly available transcriptional profiles for MTB genes (8,9). The microarray data were integrated with ∼250 000 functional gene associations from STRING (15) and nearly 5000 operon prediction associations from MicrobesOnline (21). These protein interactions and functional associations were used as input for the cMonkey algorithm to identify sets of genes that are co-expressed over subsets of environmental conditions and share *cis*-regulatory motifs in their promoters (Figure 1A) (12). Before biclustering and model construction, 77 genes (∼2%) that did not show significant expression changes or had large amounts of missing expression values were filtered out as described previously (12). Similarly, two microarray experiments were removed because of numerous missing expression values, resulting in 2325 experiment conditions that were included in the biclustering process (Supplemental Data file S1). In total, cMonkey grouped the remaining 3922 MTB genes into 598 biclusters (Supplemental Data file S2). Of note, the algorithm was set to group genes into multiple biclusters, allowing the discovery of different regulatory programs for the same gene. Out of the 598 biclusters, 444 were of high quality, with mean residual values <0.55. The residual is a metric for gene co-expression among bicluster member genes, and was calculated as previously described (12). Biclusters with ‘tighter’ co-expression profiles had a mean bicluster residual <0.55 and were passed into the high-confidence filtered network (Table 1). The residual cutoff was set at 0.55 to be more inclusive of biclusters than the EGRIN model for *Halobacterium salinarum* (12), but still maintain high statistical significance (*P*-value = 1 × 10^−5^) for the co-expression of genes in a bicluster as opposed to random genes. During biclustering, co-regulated genes were also determined based on the *de novo* detection of motifs by cMonkey (12). Motifs with an *e*-value <1 were included in the high-confidence filtered network (Table 1) (22). Altogether, cMonkey incorporated 3922 genes into the EGRIN model, resulting in the most comprehensive transcriptional regulatory network of MTB to date (Table 1) (2–3,13). Even the high-confidence filtered network that used progressively more stringent criteria (residual < 0.55 or residual < 0.55 *and* motif *e*-value < 1) had gene coverage of 84 and 55%, respectively (Table 1). To our knowledge, this is still greater coverage than that of all previously reported transcriptional regulatory networks of MTB. Unlike other models, this regulatory network provides environmental context for co-regulation of genes, along with several independent streams of evidence for the co-regulation, such as shared *cis*-regulatory motifs within gene promoters, and diverse kinds of functional associations.

**Table 1.** Summary of properties for the EGRIN model of MTB

Property	Mtb total network	Mtb filtered network
Biclusters	598	444^a^
*cis*-regulatory motifs	1192	475^b^
Gene coverage (%)	98	84^c^/55^d^

^a^biclusters with residual ≤ 0.55

^b^motifs with *e*-value ≤ 1

^c^genes in biclusters with residual ≤ 0.55

^d^genes in biclusters with residual ≤ 0.55 and *e*-value ≤ 1

### Genome-wide validation of modular architecture and regulatory mechanisms captured by the MTB model

We tested the MTB EGRIN model trained on the compendium of 2325 microarray experiments against data from newly conducted experiments that (i) mapped genome-wide DNA-binding locations of ∼200 MTB TFs and (ii) probed global transcriptional consequences of overexpressing each TF, one at a time. These two ‘TF overexpression datasets’ were not used to generate the EGRIN model and were used to assess accuracy of model predictions (Figure 1B). The premise of this test was that if the modular architecture inferred by our methodology was accurate, then there would be significant concordance between grouping of genes within modules, distribution of TF-binding locations, and the global consequences of overexpressing TFs, which would directly and indirectly alter the expression of co-regulated genes.

FLAG-tagged TFs were episomally overexpressed using a mycobacterial tetracycline-inducible promoter under standard laboratory culture conditions. Clearly, there are logistical challenges in performing overexpression experiments with over 200 strains across diverse environmental conditions. This underscores the value of network inference using published gene expression data from the wild-type strain subjected to diverse environmental conditions. Furthermore, even though the TF overexpression data were assayed in conditions that did not perfectly match conditions used to generate the training data, they proved to be extremely useful for evaluating the accuracy of the EGRIN model.

- *Dataset I: a genome-wide TF–DNA interaction map for MTB*. To map TF binding locations, ChIP-Seq was performed (2). The ChIP-Seq methodology accurately identified previously characterized TF-binding sites and was able to resolve TF binding sites with single-nucleotide resolution (23). From ∼16 000 total binding sites, we identified a total of 7248 promoter-proximal binding sites (2520 unique genes) for 143 TFs in regions spanning −150 to +70 nucleotides around transcriptional start sites across the entire genome.
- *Dataset II: global transcriptional consequences of overexpressing ∼200 TFs in MTB*. We analyzed RNA from the same cultures in which TFs were induced for ChIP-Seq to assay global transcriptional consequences of overexpressing each of 206 TFs. We identified 3785 unique mRNAs of significant expression change (*P*-value < 0.01).

To integrate these datasets and employ them for validation, we first investigated how often two or more genes were simultaneously within a bicluster, bound by the same TF and differentially regulated upon over-expression of that TF. This analysis identified a set of 454 unique genes that were co-regulated in varying combinations across 240 biclusters by 57 TFs (Benjamini–Hochberg, BH, corrected permuted *P*-value < 0.01, Supplemental Data file S3). We also compared the genes in biclusters discovered by EGRIN to the experimentally characterized targets of every overexpressed TF. This comparison showed that the network model accurately recalled co-regulated genes for 41% of the overexpressed TFs (57 out of 140 at *P*-value ≤ 0.05 for all TFs with ≥2 unique genes) and recovered 49% of the TF–gene interactions from the TF overexpression set (793 out of 1635 genes that were both ChIP-Seq targets and differentially expressed upon over-expression of a TF). The 49% recovery rate is greater than validated interactions from other transcriptional regulation modeling algorithms using expression data (24,25). Notwithstanding the successful validation of model predictions by this stringent analysis, it should be noted that this test is restricted to validating regulatory mechanisms that are active in the context in which the physical interactions and overexpression consequences were assayed. The gene regulatory network model encompasses a much larger set of regulatory mechanisms that are active across diverse environmental conditions, which underlies a key capability of the EGRIN model, i.e., to make predictions of conditional regulation across environments. Indeed, a larger set of 671 modules contained two or more genes that shared binding sites for a TF (BH corrected hypergeometric *P*-value < 0.01). The 671 modules take into account redundant modules that can be associated with more than one TF. We also compared the *cis*-regulatory motifs of biclusters to motifs that were deciphered through analysis of ChIP-Seq mapped binding locations. Bicluster motifs with significant matches to at least one TF motif (False Discovery Rate, FDR, *q*-value < 0.01) are shown in Supplemental Data file S4. Overall, the model predicts that 1117 unique interactions for 113 TFs in the P–D interaction network are potentially functional in some environmental context included in the gene expression compendium used for model construction. Similarly, a larger set of 261 redundant biclusters was enriched for two or more genes that were differentially expressed upon over-expression of a TF (BH corrected hypergeometric *P*-value < 0.01), validating regulation of 1062 unique genes by 137 TFs (Figure 1B). The context in which genes are grouped together in the bicluster also predicts an appropriate environmental context in which to map P–D interactions of a particular TF, possibly justifying the added effort (such as chromosomal tagging strategy, or generating an antibody against the native protein) required to overcome technical challenges in mapping functional interactions of that TF. Thus, it is impressive that co-regulation of genes across 240 biclusters could be validated just with experiments performed under standard growth conditions. Moreover, we compared the validated bicluster targets of two TFs, DosR and KstR, to their gene targets reported in transcriptional regulatory networks of hypoxia (3) and macrophage infection (5) and found significant overlap (*P*-value < 0.01), despite the different experimental conditions and modeling methods used. Importantly, the gene regulatory network model presented here makes predictions of various environmental contexts, beyond hypoxia and macrophage infection, in which genes are co-regulated by specific TFs.

### Insights into contextual regulation of specific functions in MTB

Mechanistic features of the gene regulatory network were validated by comparisons to the independently generated TF–DNA interaction map and global transcriptional consequences of overexpressing TFs. Next, we investigated how the gene regulatory network could be used to gain insight into specific environment-induced dynamic changes in MTB physiology. Statistical analysis for enrichment of GO terms (6) revealed that co-regulated genes within 33 of the 179 validated biclusters (18%) from the high-confidence filtered network were associated with functional GO categories (Supplemental Data file S3). Given the nature of the experiments within the gene expression compendium, it is not surprising that the most common functional category represented among the co-regulated genes was ‘growth of symbiont in host’. The conditional grouping of genes within biclusters further provides an opportunity to elucidate the context in which these functionally related genes were co-regulated. In other words, while the TF overexpression datasets validated functional interactions within the gene regulatory network, only a subset of these interactions would be active, and biologically meaningful in specific environments.

**Figure 2.**
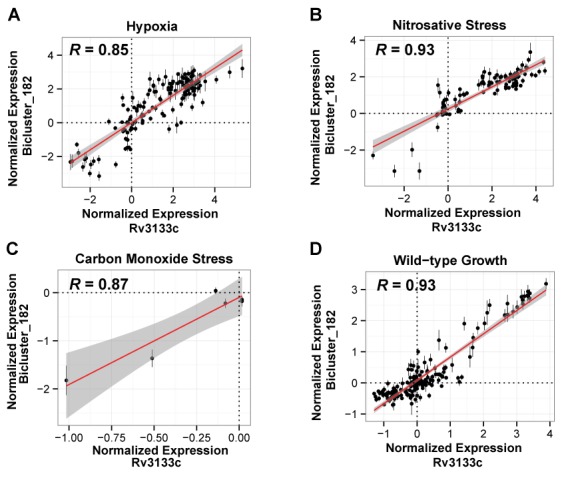
The conditional regulation of bicluster_182 by DosR. The scatter plots show the correlation of expression for DosR versus the median correlation of gene members of bicluster_182 under (**A**) hypoxia (*R* = 0.85, *P*-value = 0.004), (**B**) nitric oxide (*R* = 0.87, *P*-value < 2 × 10^−16^), (**C**) carbon monoxide stress (*R* = 0.87, *P*-value = 0.0001) and (**D**) wild-type growth conditions (*R* = 0.93 and *P*-value < 2 × 10^−16^). Error bars show the standard deviation of bicluster gene expression. The number of data points in each plot equals the number of transcriptome profiles in the environmental context shown in the plot.

We hypothesized that the context in which expression of a TF is significantly correlated to its target genes would provide evidence of condition-specific regulation. We investigated patterns of correlations between TFs and their validated bicluster genes (from biclusters in the filtered network) across 1355 transcriptome profiles from the compendium data (see ‘Materials and Methods’ section for selection criteria, Supplemental Data file S5), classified into 14 environmental contexts (re-aeration, nitrosative stress, hypoxia, etc.). Based on this method, 46 TFs were significantly correlated or anti-correlated to their validated bicluster target genes across environmental contexts (Pearson's correlation coefficient −1.0 ≤ *R* ≥ −0.85 or 0.85 ≤ *R* ≥ 1.0 and *P*-value ≤ 0.05; Supplemental Data file S6 and Supplementary Figure S1). As a specific example, Figure 2A shows that expression changes in DosR (Rv3133c) were correlated with co-regulated genes within bicluster_182, over 150 experiments that were conducted under hypoxic conditions. Remarkably, the gene regulatory network has reconstructed *de novo* the DosR regulon including 31 of the 49 genes (BH corrected hypergeometric *P*-value = 2.14 × 10^−5^) that are known to be induced by hypoxia, nitric oxide and redox stress (26). Independently, the gene regulatory network predicted that DosR conditionally co-regulates these genes under hypoxia (*R* = 0.85, *P*-value = 0.004), nitric oxide (*R* = 0.87, *P*-value < 2 × 10^−16^) and carbon monoxide stress (*R* = 0.87, *P*-value = 0.0001, Figure 2A–C) (26–31). Additionally, the network predicted that the DosR regulon is correlated under standard growth conditions (*R* = 0.93 and *P*-value < 2 × 10^−16^) (Figure 2D). This result potentially explains why this regulon was significantly perturbed upon overexpression of DosR under standard growth conditions (2). The network model also predicted new environmental conditions in which the DosR regulon was active such as interactions with host surfactants. Using the contextual prediction of validated biclusters, we also identified 183 genome-wide regulatory mechanisms that act uniquely during host infection (i.e., growth on cholesterol, host interactions and growth in macrophage), but were not observed under normal laboratory growth conditions (Supplemental Data file S7), predicting some key factors related to host–pathogen interactions. Overall, the ability of the gene regulatory network to predict both the composition of a regulon and the context in which it is active provides the information required (genetic background of strain, growth conditions and phenotypic assay) to facilitate experimentation for iteratively testing and refining the model.

### Insights into transcriptional regulation of cholesterol utilization genes

The utilization of host-derived cholesterol has attracted considerable attention since the discovery of its important role in MTB virulence and persistence in the host (7). During persistence in the host, cholesterol serves as an essential nutrient source as well as a precursor molecule for the biosynthesis of complex fatty acids (7,32). Moreover, deletion of MTB genes involved in cholesterol utilization has been shown to attenuate infection outcome (7,32–35). We saw opportunity to use the network model to delineate mechanisms and context for regulation of cholesterol utilization genes, which, despite the importance of this process, remain poorly understood. This study was also motivated and facilitated by a previously conducted genome-wide screen on essentiality of genes for cholesterol utilization in MTB (18).

**Figure 3.**
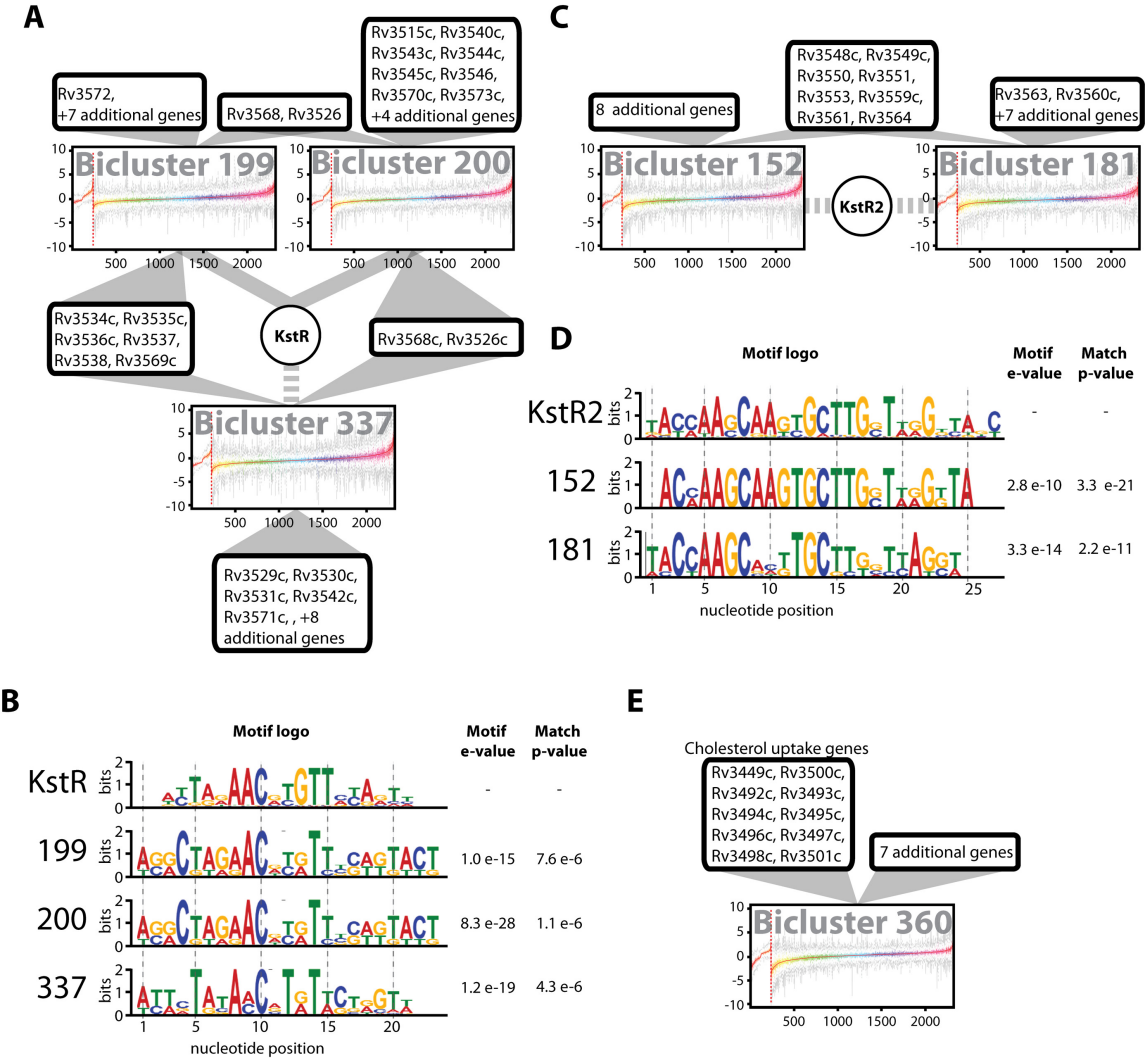
The regulatory interaction subnetwork of cholesterol utilization. The figure shows a subset of biclusters enriched for genes essential for growth on cholesterol (see text). (**A**) *KstR* is a gene member of bicluster 199 and bicluster 200, as shown by solid lines. Bicluster 337 was associated with KstR through motif analysis (see text), which is represented by dashed lines. Genes involved in cholesterol utilization are listed and connected by solid lines to their regulatory biclusters. (**B**) The motif logos detected for KstR and biclusters from panel (A). The *e*-values of the motifs and *P*-values from alignment with KstR are shown. (**C**) Biclusters involved in cholesterol utilization and associated with KstR2 by motif analysis. (**D**) The motif logos detected for KstR2 and biclusters from panel (C). The *e*-values of the motifs and *P*-values from alignment with KstR2 are shown. (**E**) Bicluster 360 contains 10 genes that are involved in cholesterol uptake.

We discovered that six biclusters within the gene regulatory network were significantly enriched for genes essential for *in vitro* growth on cholesterol (BH corrected hypergeometric *P*-value < 0.01, Supplemental Data file S8). Among these six biclusters, three modules (bicluster_199, bicluster_200, bicluster_337) were dominated by genes that degrade the side-chain and rings A/B of the cholesterol molecule (Figure 3A) (36–39). Together, these three biclusters contain 22 genes that are essential for growth on cholesterol, and all were enriched for genes annotated with functions in metabolism and catabolism of organic steroid small molecules. More specifically, a significant number of genes within the biclusters had ChIP-seq mapped binding sites for KstR (Rv3574) and were differentially expressed upon overexpression of this known TetR family regulator (40) of cholesterol utilization in MTB (BH corrected hypergeometric *P*-value < 0.01). Analysis of *de novo* detected *cis*-regulatory motifs within bicluster_199, bicluster_200 and bicluster_337 using TOMTOM (a database of prokaryotic regulatory motifs, Reg TransBase v4) (19) followed by a Basic Local Alignment Search Tool (BLAST) search of the MTB genome revealed an almost perfect match to the known motif for KstR (*P*-values < 0.01, Figure 3B) (40). Supplemental Data file S4 also shows the motifs of bicluster_199 (*q*-value = 6.6 × 10^−3^) and bicluster_200 (*q*-value = 1.8 × 10^−6^) were independently discovered through analysis of the ChIP-Seq binding locations for KstR. In addition to accurately reconstructing the KstR regulon, the gene regulatory network model also predicted that it is active under cell envelope stress, hypoxia (41) and oxidative stress conditions (Supplemental Data file S5, Figure S2) as well as growth in standard laboratory conditions (data not shown).

**Figure 4.**
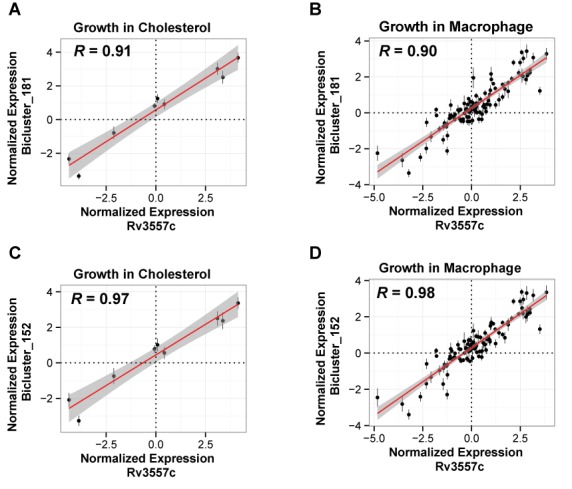
The conditional regulation of KstR2 biclusters. The scatter plots show the correlation of expression for KstR2 versus the median correlation of gene members of bicluster_181 under (**A**) growth on cholesterol (*R* = 0.98, *P*-value = 5.6 × 10^−6^) and (**B**) growth in macrophage (*R* = 0.90, *P*-value < 2 × 10^−16^) and KstR2 versus gene members of bicluster_0152 under (**C**) growth on cholesterol (*R* = 0.97, *P*-value = 9.0 × 10^−6^) and (**D**) growth in macrophage (*R* = 0.91 and *P*-value < 2 × 10^−16^). Error bars show the standard deviation of bicluster gene expression. The number of data points in each plot equals the number of transcriptome profiles in the environmental context shown in the plot.

From the six biclusters in the cholesterol utilization subnetwork, two biclusters (bicluster_152 and bicluster_181) were enriched for 10 out of the 15 genes (BH corrected hypergeometric *P*-value = 3.10 × 10^−21^) reported to catabolize the rings C/D of cholesterol (Figure 3C) (42,43). Genes within these biclusters were enriched for ChIP-Seq mapped binding sites (BH corrected hypergeometric *P*-value < 0.01) of another TetR-type transcriptional regulator, KstR2 (Rv3557c) (44). Again, the cMonkey detected motifs of bicluster_152 and bicluster_181 that were significantly similar to the known KstR2 motif (*P*-values < 0.01, Figure 3D) (44). Notably, the KstR2-regulated biclusters also had an over-representation of genes for ‘growth of symbiont in host’ (*P*-value < 0.0026), suggesting that KstR2 coordinates cholesterol utilization with other genes essential for proliferation within macrophages. Consistent with this observation, expression changes of KstR2 were maximally correlated (*R* > 0.90, *P*-value < 9.0 × 10^−6^) with corresponding changes in its putative target genes of bicluster_152 and bicluster_181 in experiments that had independently probed growth using cholesterol (36) and within macrophages (Figure 4A–D) (45–49). Interestingly, the 183 regulatory mechanisms that act uniquely during host infection were significantly enriched (23 genes, BH corrected hypergeometric *P*-value < 0.01) in genes essential for growth on cholesterol (19) (Supplemental Data file S7) and specifically those in biclusters regulated by KstR2 (11 genes, BH corrected hypergeometric *P*-value < 0.01). This suggests the importance of KstR2 in regulating factors involved in host–pathogen interactions. It also emphasizes the value of our model to predict regulatory interactions that occur in important contexts (i.e., pathogenesis) that would otherwise be missed in standard laboratory experimentation.

All 10 genes for cholesterol uptake including the ABC transporter Mce4 (50), were grouped together in bicluster_360 (Figure 3E). Contrary to previous reports, there was no evidence in the gene regulatory network that the Mce4 genes were regulated by KstR or KstR2 (51). None of these genes had ChIP-Seq-mapped binding sites for either of the two TFs, nor did the *cis*-regulatory motifs within bicluster_360 align with KstR or KstR2 motifs. An *in silico* approach and a promoter pull-down assay also failed to identify any TFs of the Mce4 operon (52). Instead, the gene regulatory network model predicted that genes within bicluster_360, including Mce4, were regulated by Rv3575c, a Lac I family regulator. Remarkably, this regulator was discovered to be essential for growth on cholesterol, providing an independent validation of our model prediction (18).

The power of making an accurate prediction of environment-specific regulation is underscored by the fact that a very small number of experiments in the entire compendium of gene expression profiles were performed in cholesterol-rich conditions (9 out of 2325, 0.4%). Yet, the network extended our understanding of the cholesterol utilization regulatory network by revealing that cholesterol uptake is regulated by a distinct sub-network. Most importantly, the gene regulatory network was able to tease apart regulation of the three distinct components of cholesterol utilization, i.e., cholesterol uptake, degradation of A/B sterol rings and catabolism of C/D rings. The network even made accurate predictions of known regulation of A/B ring degradation by KstR and C/D ring degradation by KstR2, and furthermore it provided context for regulation of these pathways.

## CONCLUSIONS

In this study, we compiled a large compendium of 2325 gene expression profiles from different studies to reconstruct the transcriptional regulatory network for nearly all of the genes in MTB. Our model reveals environmental context-dependent co-regulation of up to 3922 genes (98%) in various combinations and provides a multi-scalar picture of gene regulation in conditions and life cycles experienced by MTB within the host organism. While the systems biology approach can be readily extended to new data types and even the host, it should be noted that in its current form, the model does not capture all types of regulatory mechanisms, such as those mediated by epigenetic, second messenger signaling (53,54) or post-transcriptional mechanisms. But the foundation laid in the process of constructing this network model has paved the way for future incorporation of new modeling techniques for overcoming these limitations (55–58). Moreover, we expect that this model will drive collaborations across the MTB community, and establish an iterative cycle of hypothesis formulation, experimental testing and model refinement. Specifically, the network model can be used to formulate experimentally testable hypotheses regarding regulation of specific genes in the MTB genome—this capability to prioritize experiments will be enormously useful given the challenges of working with this pathogen. This network can also be used to make discoveries of novel functional associations across genes of known, but previously unrelated, functions and even genes of previously unassigned functions. We demonstrated the mechanistic and predictive accuracy of this network model by generating a TF-DNA physical interaction map for 143 TFs in MTB, as well as a compendium of transcriptome profiles from 206 strains, each overexpressing a different TF. The network model extracted statistically significant and biologically meaningful features from both of these genome-scale complex datasets, to demonstrate unequivocally the power of this new resource for the MTB community. Using two subnetworks, one for regulation of hypoxia and the second for regulation of cholesterol utilization, we demonstrated how the network can be used to gain insights into environment-dependent regulation of specific genes, and pathways. To enable similar explorations of the new model, we have made it accessible through a user-friendly web-portal that integrates new and previously established resources for MTB (http://networks.systemsbiology.net/mtb/).

**Figure 5.**
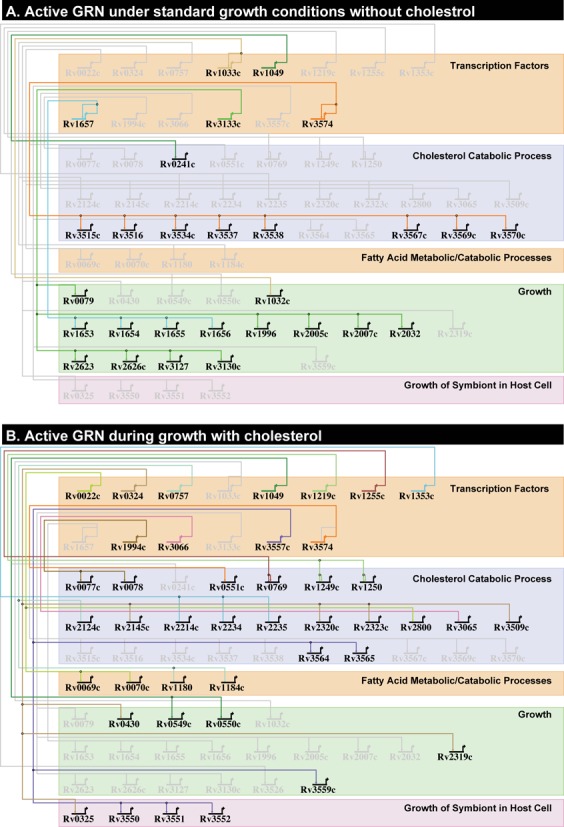
Enivronment-specific gene regulatory networks for *Mycobacterium tuberculosis.* This figure shows a subset of the gene regulatory network of MTB under (**A**) wild-type growth conditions and (**B**) growth in cholesterol, derived from BioTapestry visualization. Transcription Factors (TFs) are grouped together and represented by bent arrows, which extend to horizontal and vertical lines that connect to their regulatory gene targets. The gene targets that are shown were predicted by the EGRIN model to be co-regulated in biclusters and were validated for regulation by their linking TF through ChIP-Seq binding and differential expression from TF overexpression experiments (see text). Inclusion in these models required significant correlation of expression of TF and validated bicluster genes under wild-type growth (A) or growth in cholesterol (B). The arrows and barred lines indicate the direction of correlation (activation or repression). Only gene targets with annotated function that fall into the categories of ‘cholesterol processes’ (metabolic/catabolic), ‘fatty acid processes’ (metabolic/catabolic), ‘growth’ or ‘growth of symbiont in host cell’ are found in the regulatory networks shown here. An interactive version of the BioTapestry network model, with additional environmental contexts, can be found at the MTB Network Portal, located at http://networks.systemsbiology.net/mtb/.

The MTB Network Portal also contains an interactive BioTapestry network viewer that nicely demonstrates the regulation of specific genes in a selection of environmental conditions we investigated. The architecture of the BioTapestry network is based on the validated bicluster genes and their regulatory influences (TFs and environmental). The network is condensed to only contain genes with annotated functions that are grouped into broad categories of ‘cholesterol processes’, ‘fatty acid processes’, ‘growth’ and ‘growth of symbiont in host cell’. Due to the complexity of transcriptional regulation, a single overview network is not sufficient and this visualization method allows for an easy comparison between environmental contexts. By just comparing the regulatory interactions that are regulated during wild-type growth to those that are regulated during growth on cholesterol, it is possible to glean some interesting biological insights (Figure 5). In particular, we noticed that only two TFs regulate genes in both conditions, those being Rv3574/KstR and Rv1049. We discussed previously the regulatory activity of KstR under various conditions. However, it is interesting that during growth on cholesterol, Rv1049 would transition from activating a gene associated with cholesterol processes to activating genes involved in wild-type growth. Interestingly, the Rv1049 activated genes during growth on cholesterol are *vapBC3* (Rv0549–Rv0550), which encode a type II toxin-antitoxin (TA) system (59). MTB possesses a large number of TA systems in its chromosome, 79 in total, and *vapBC3* was among 10 significantly up-regulated in persistent cells (60). It is thought that in response to environmental cues, active toxins are capable of inhibiting DNA translation or degrading mRNA, thus allowing MTB to enter a slow or non-replicating state and establish latent infection (61). Moreover, a recent study in *Mycobacterium smegmatis* showed that control of carbon (sugar) utilization was mediated by the only TA system in *M. smegmatis*, VapBC (62). They demonstrated that VapC is an RNase that targets specific mRNA transcripts involved in carbon transport and metabolism, particularly those involved in glycerol metabolism (62). This suggests TA systems are capable of fine-tuning metabolic processes in *Mycobacteria* at a posttranscriptional level. Therefore, it is interesting to hypothesize that in response to growth on cholesterol, Rv1049 up-regulates the transcription of VapBC3, and that toxin VapC3 degrades mRNA transcripts involved in the uptake and metabolism of carbon sources present during wild-type growth, i.e., glycerol. The degradation of glycerol/sugar uptake and metabolism transcripts would prevent the accumulation of excess sugar phosphates and allow the bacteria to transition to the machinery needed for growth on cholesterol. In this way, it is possible that VapBC3, and in turn Rv1049, control cholesterol utilization in MTB and meet the demands of the various nutrient sources available to MTB during infection in the host. This is just one of many novel biological predictions that can be made from our environment-specific transcriptional regulatory network of MTB.

## SUPPLEMENTARY DATA

Supplementary Data are available at NAR Online.

SUPPLEMENTARY DATA
