# Supplementary material for: A high-resolution network model for global gene regulation in Mycobacterium tuberculosis
Source: Nucleic Acids Res. 2014 Sep 17;42(18):11291–303. doi: 10.1093/nar/gku777 (PMC4191388; doi:10.1093/nar/gku777)
Supplement: SUPPLEMENTARY DATA [file supp_42_18_11291__index.html]

A high-resolution network model for global gene regulation in Mycobacterium tuberculosis — A high-resolution network model for global gene regulation in Mycobacterium tuberculosis — SUPPLEMENTARY DATA 

# A high-resolution network model for global gene regulation in *Mycobacterium tuberculosis*

## SUPPLEMENTARY DATA

**Files in this Data Supplement:**

- SUPPLEMENTARY DATA
