# Supplementary material for: A high-resolution network model for global gene regulation in Mycobacterium tuberculosis
Source: Nucleic Acids Res. 2014 Sep 17;42(18):11291–303. doi: 10.1093/nar/gku777 (PMC4191388; doi:10.1093/nar/gku777)

Supplemental data file S6.

Figure S1. The conditional regulation of TFs and biclusters. The scatter plots show the correlation of expression for TFs versus the median correlation of gene members of the indicated bicluster under particular environmental conditions. The pearson's correlation coefficient (R) and *p*-value (*p*) are indicated for each plot. Error bars show the standard deviation of bicluster gene expression. Only correlations that meet the criteria detailed in the text are included here.

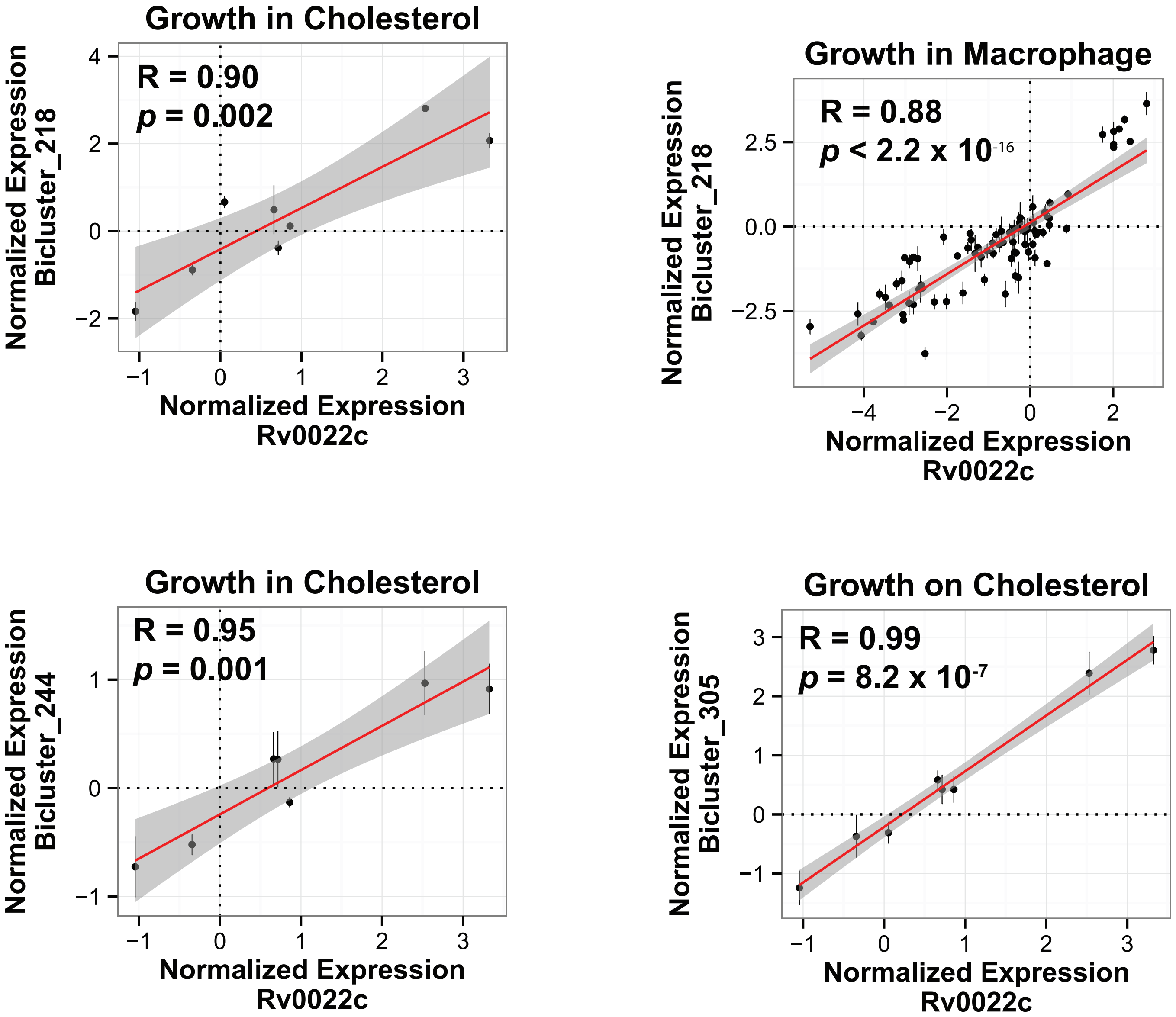

Supplemental data file S6.

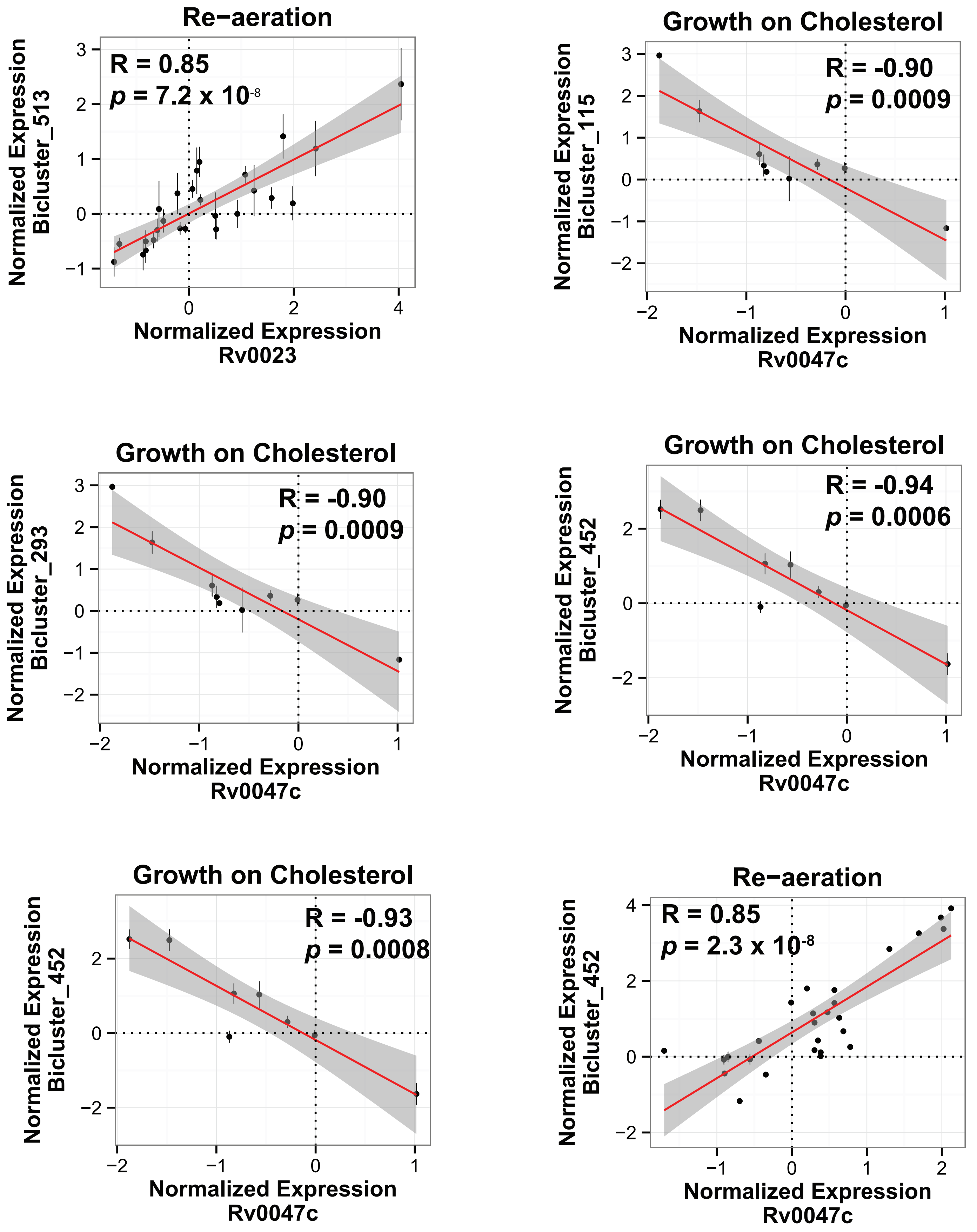

Supplemental data file S6.

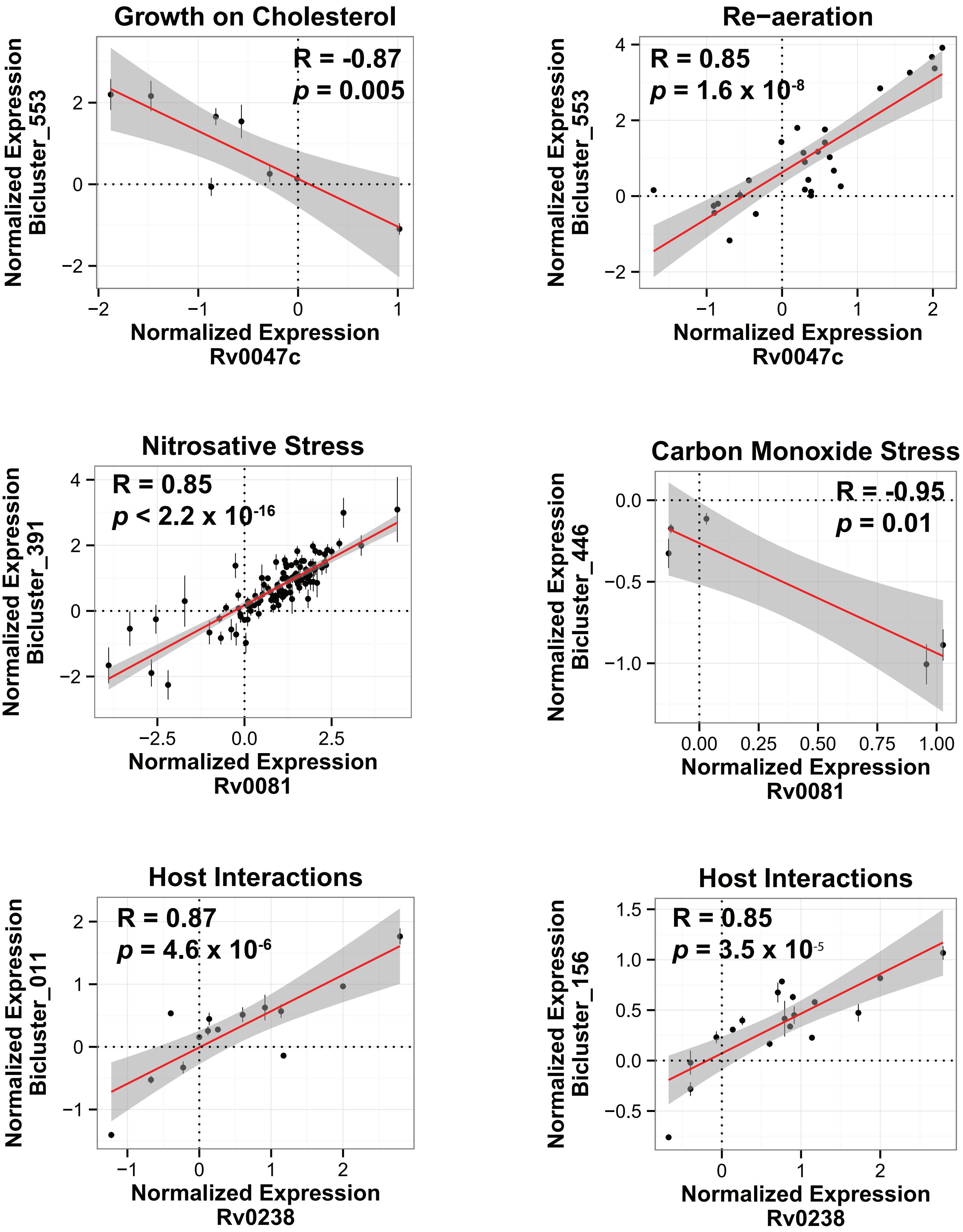

Supplemental data file S6.

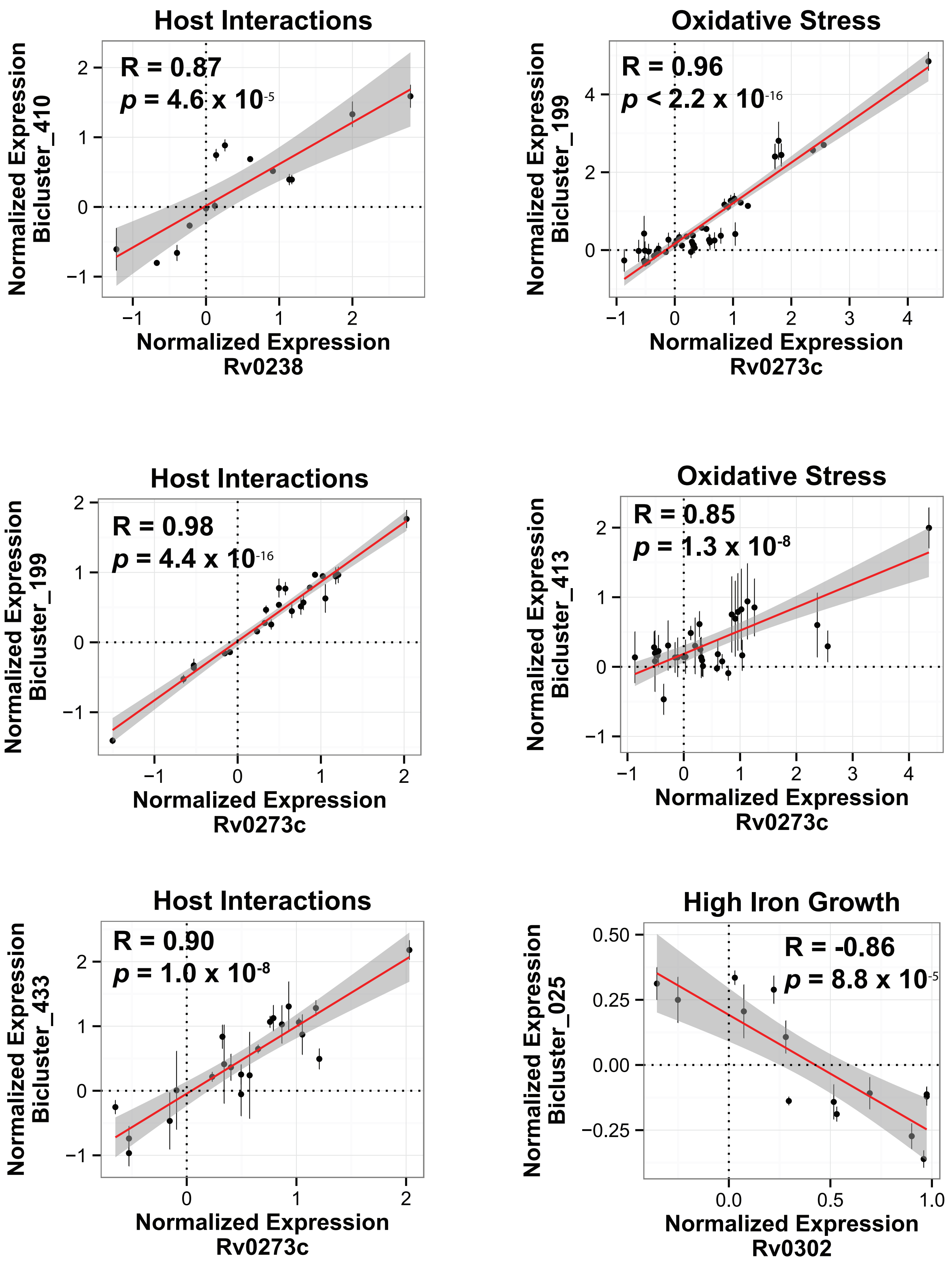

Supplemental data file S6.

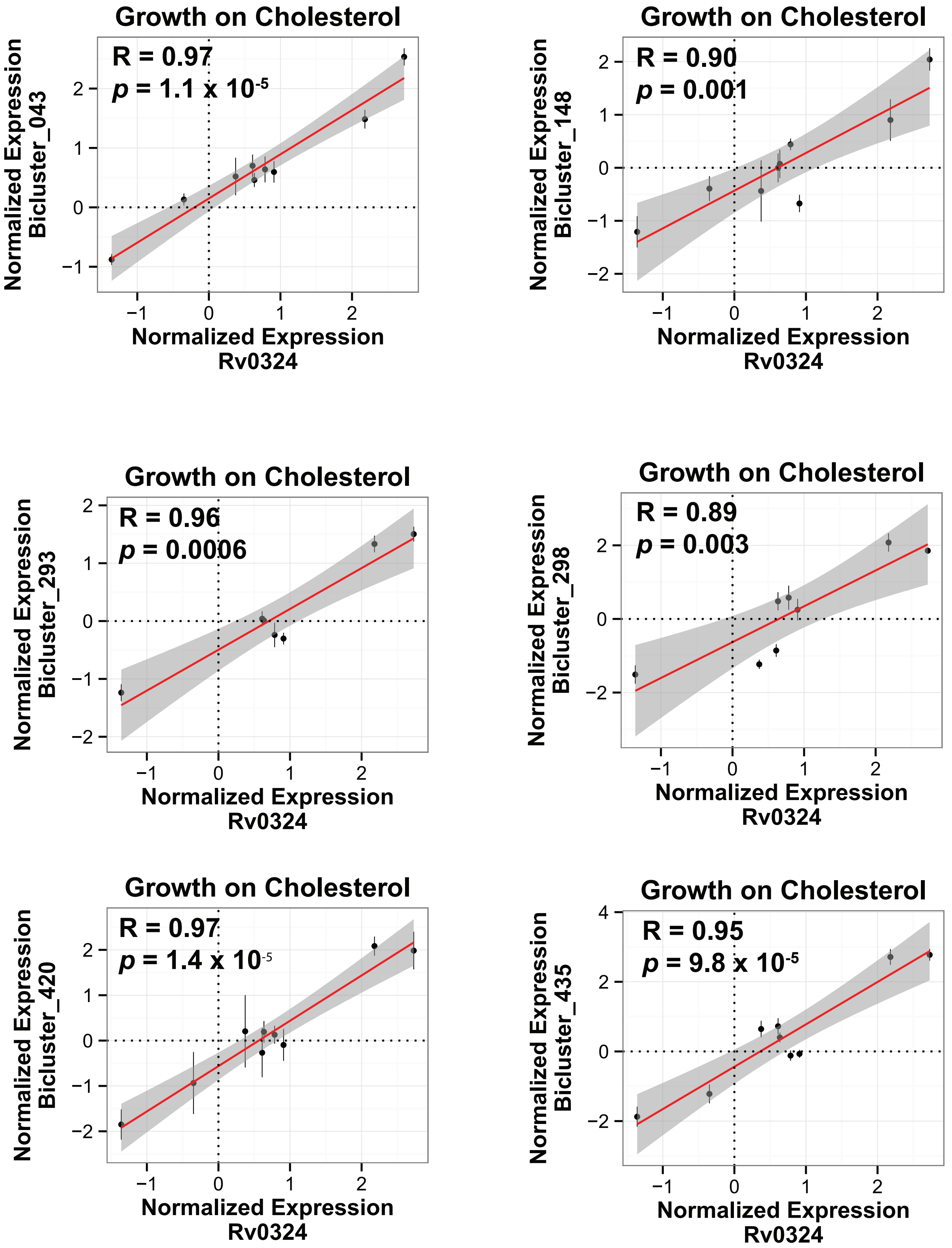

Supplemental data file S6.

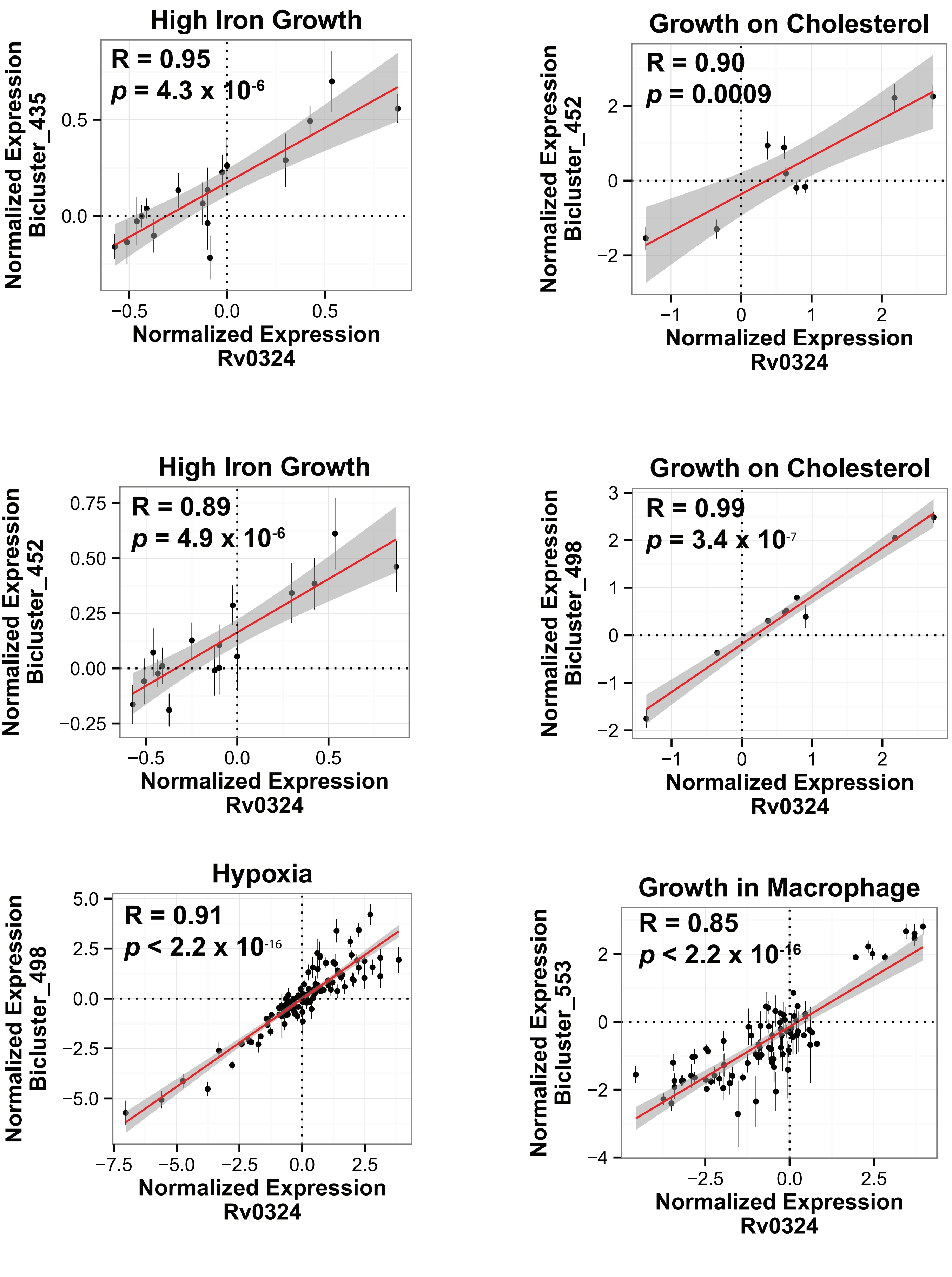

Supplemental data file S6.

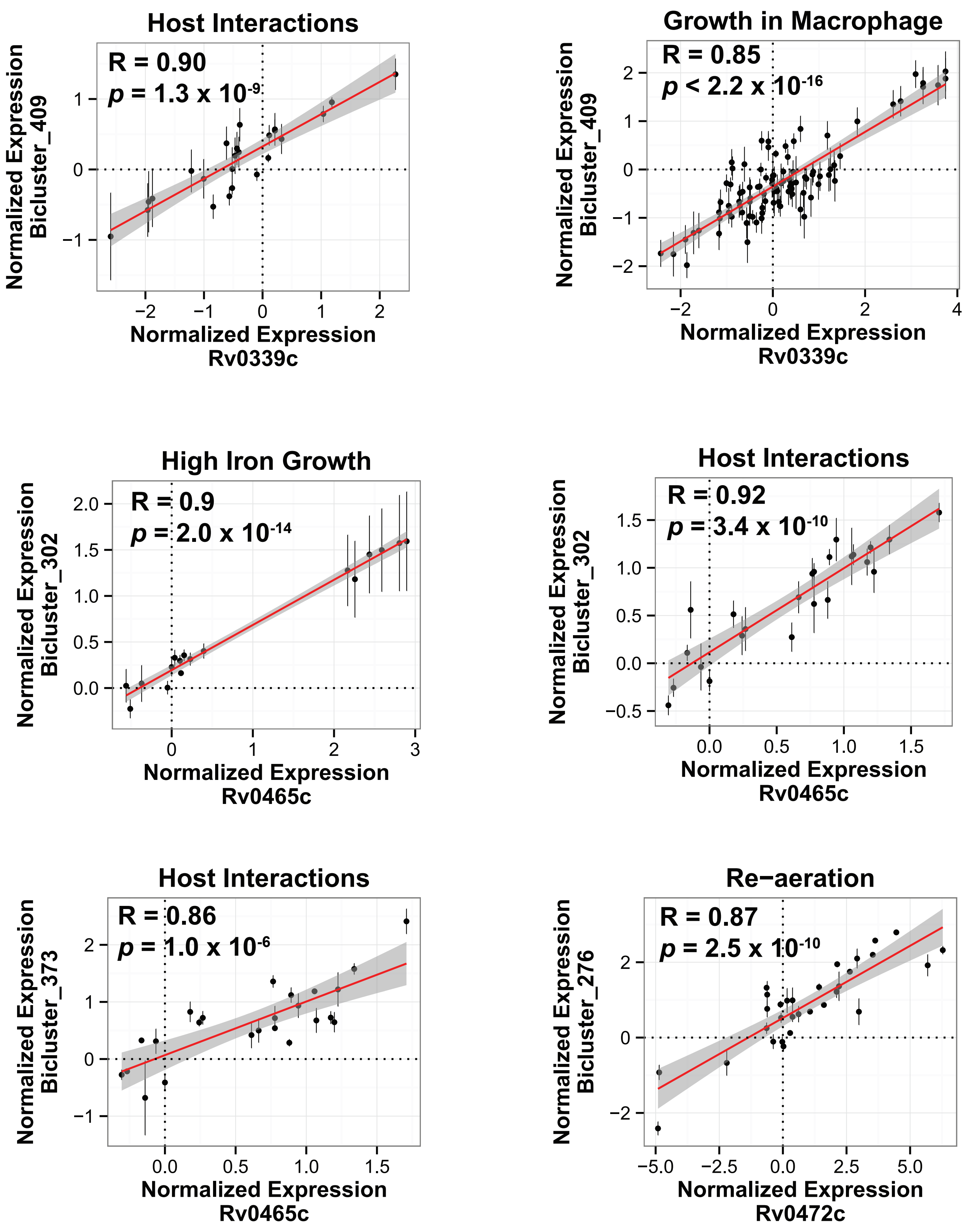

Supplemental data file S6.

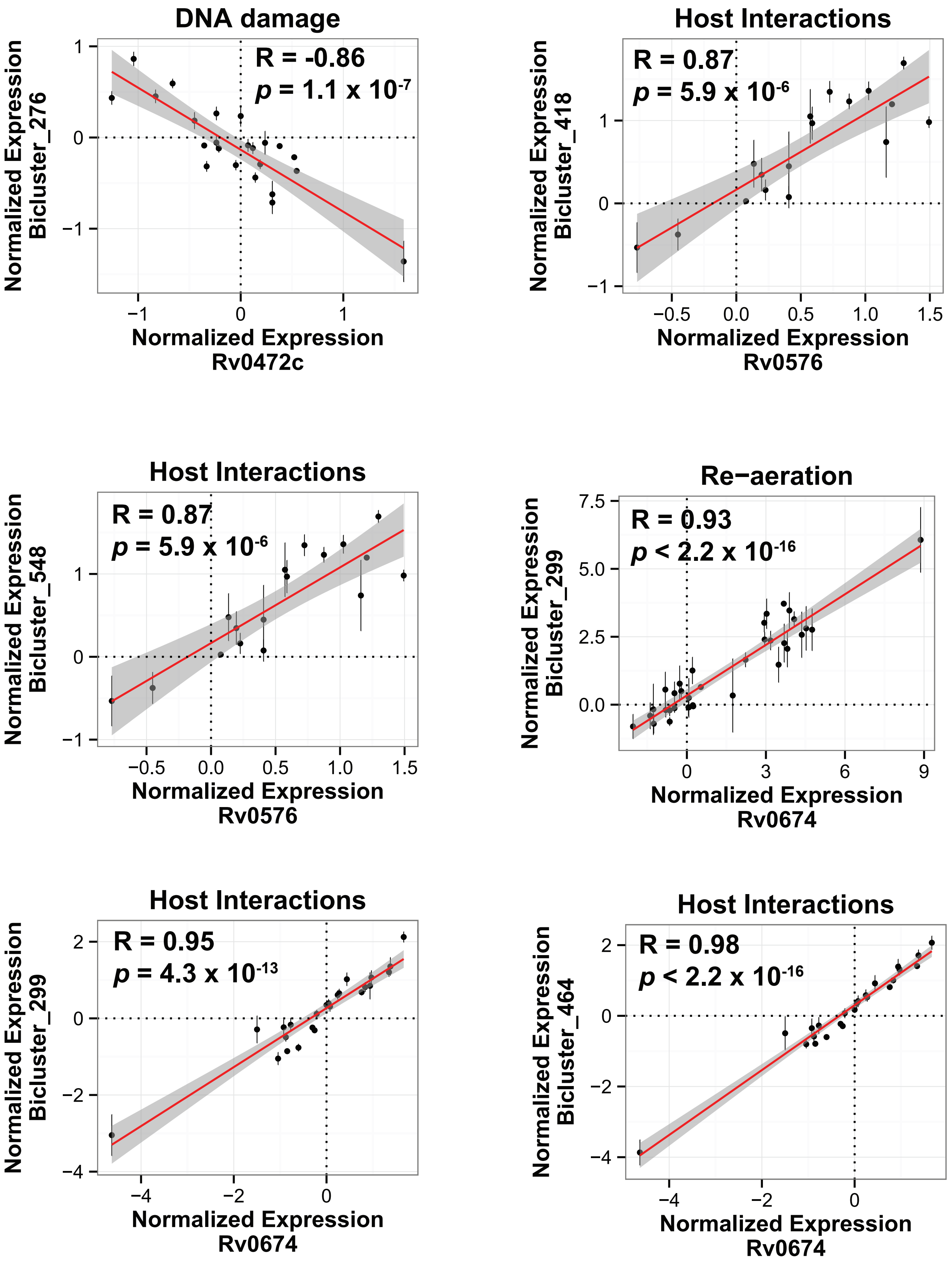

Supplemental data file S6.

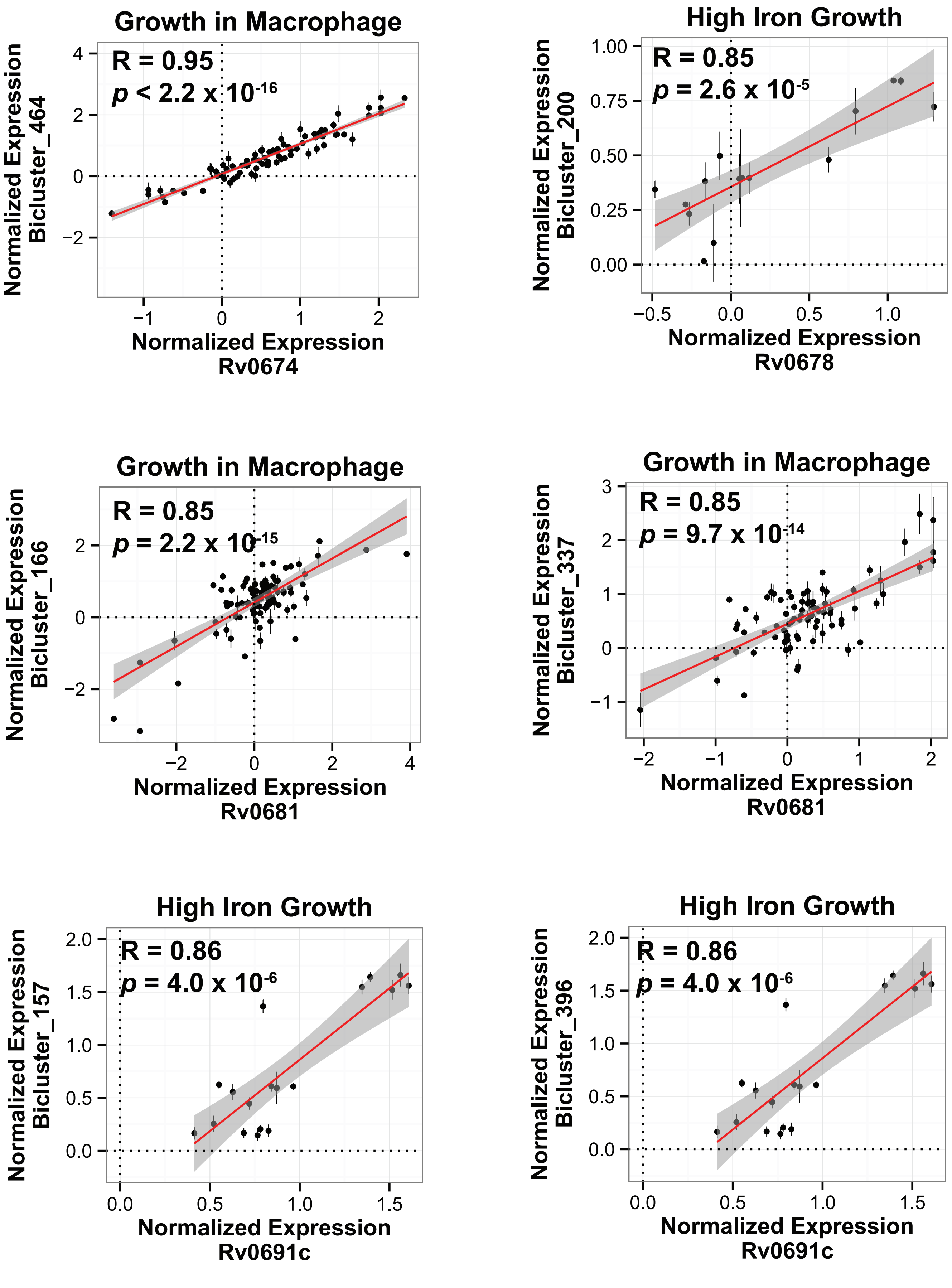

Supplemental data file S6.

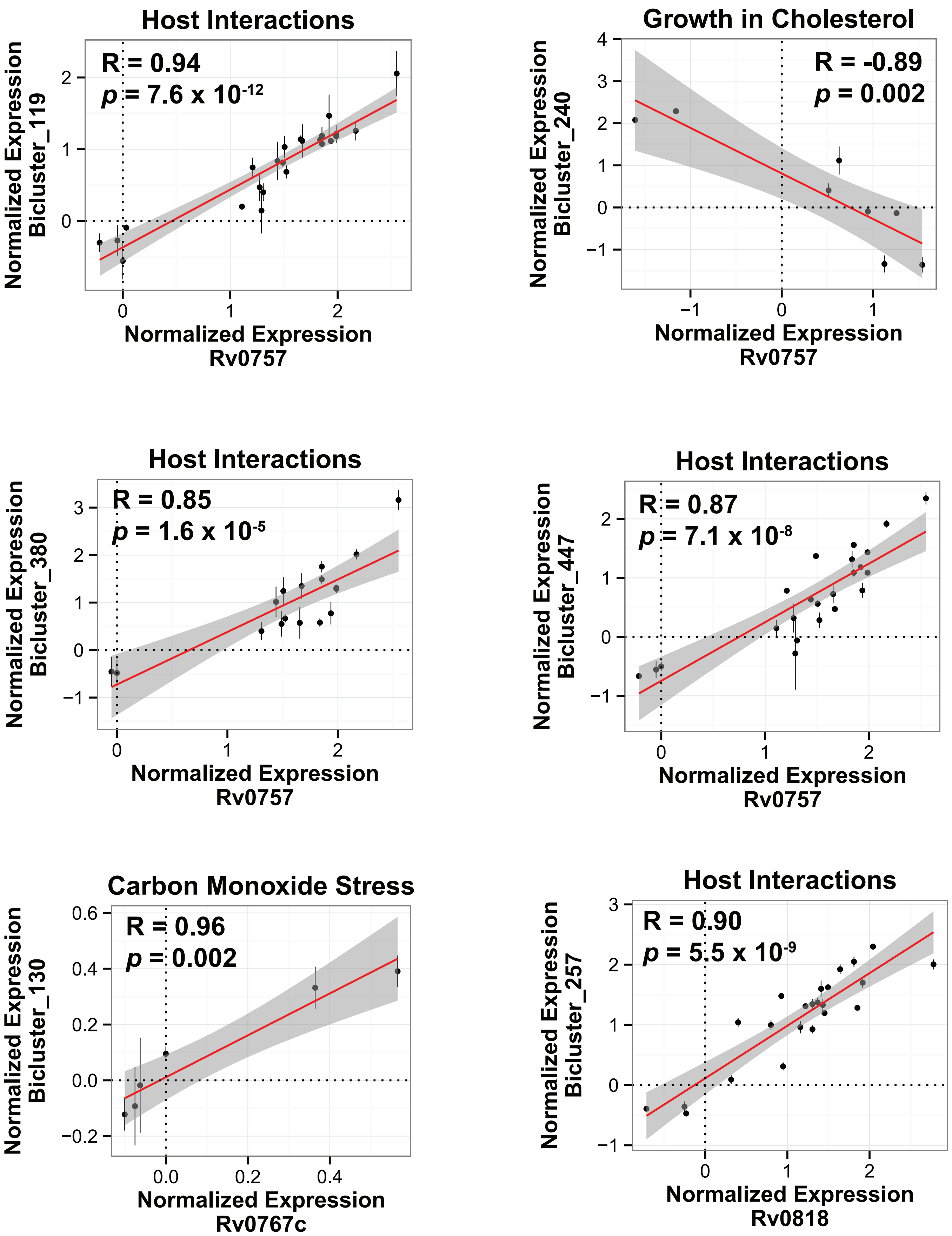

Supplemental data file S6.

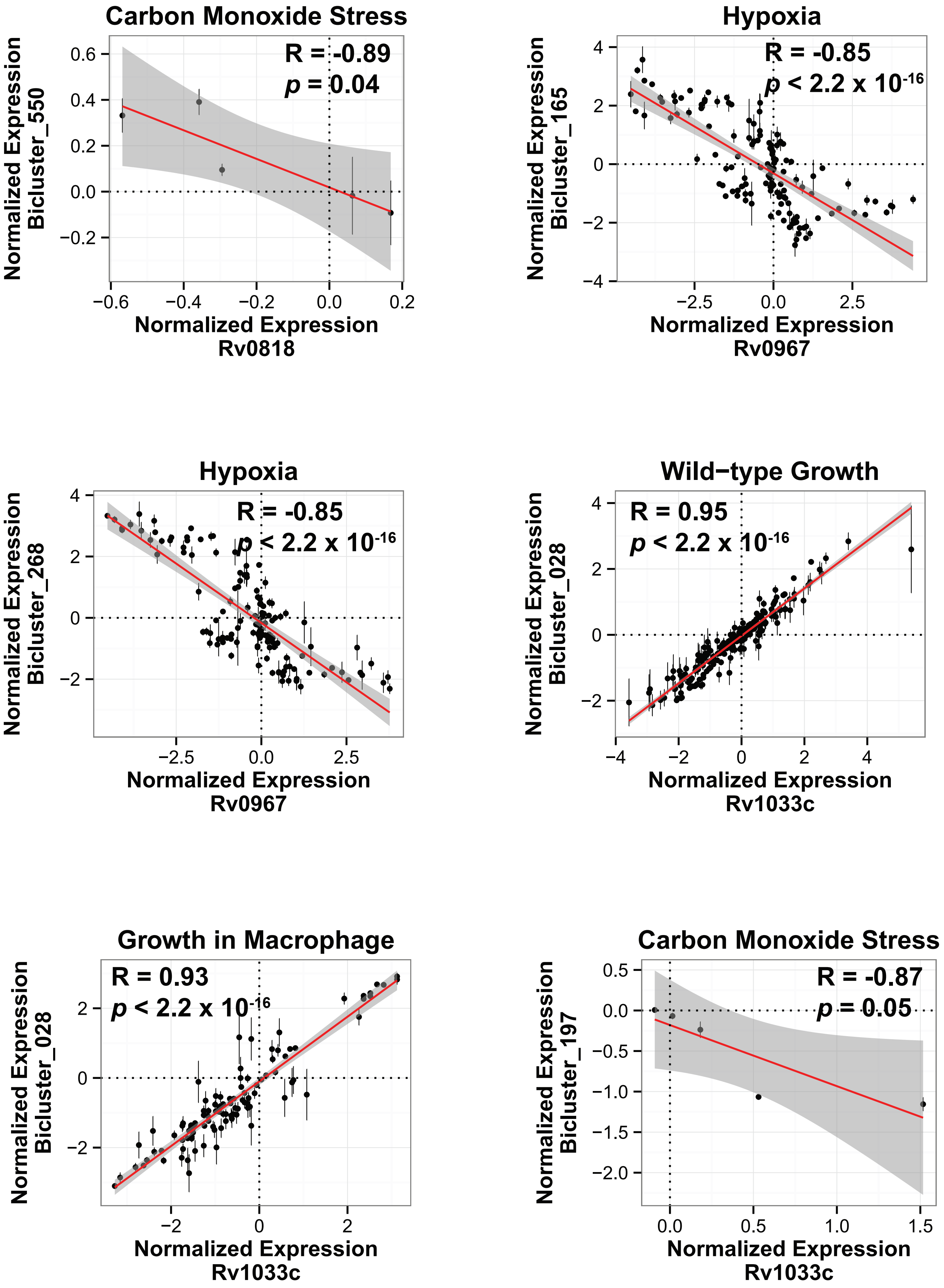

Supplemental data file S6.

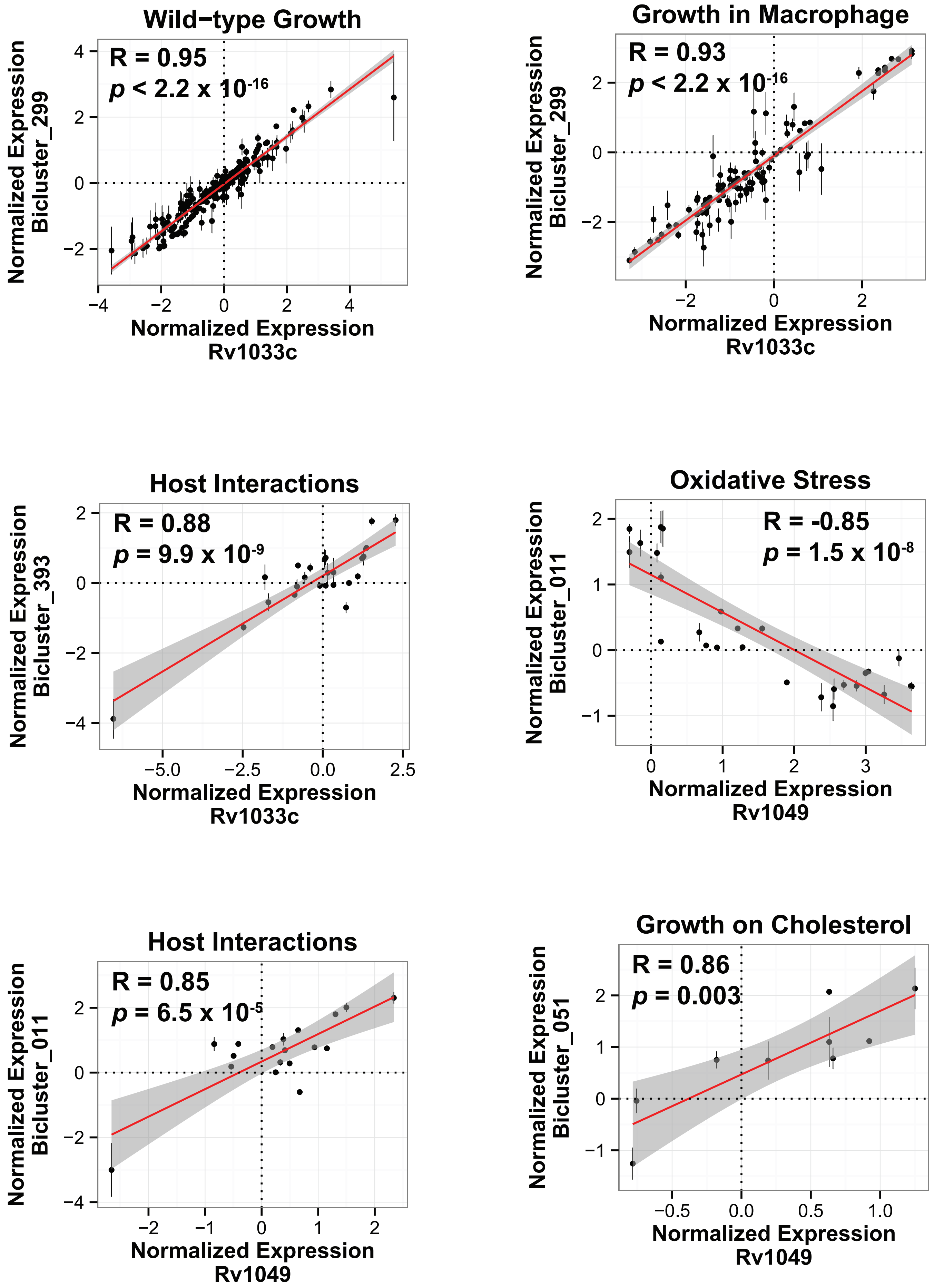

Supplemental data file S6.

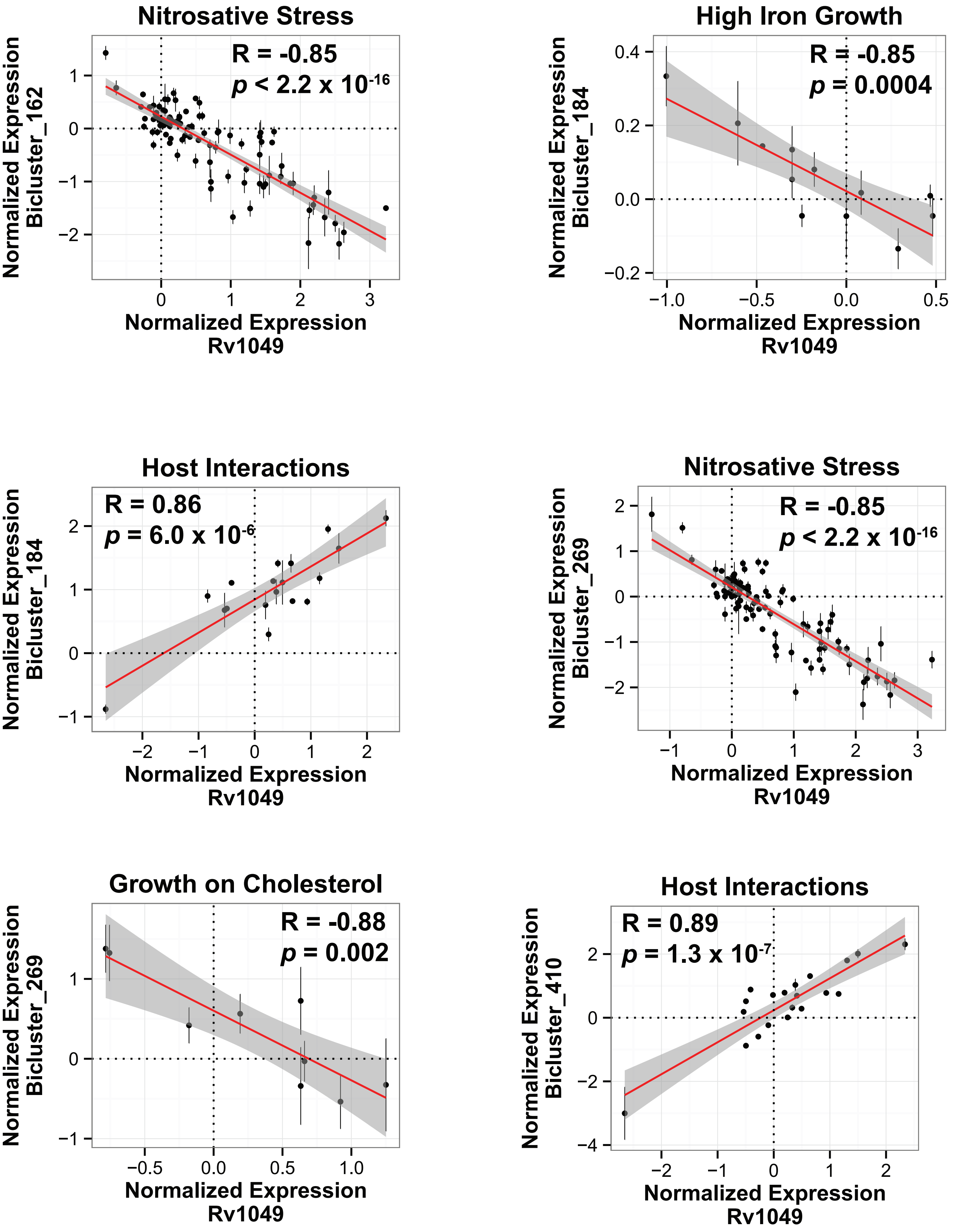

Supplemental data file S6.

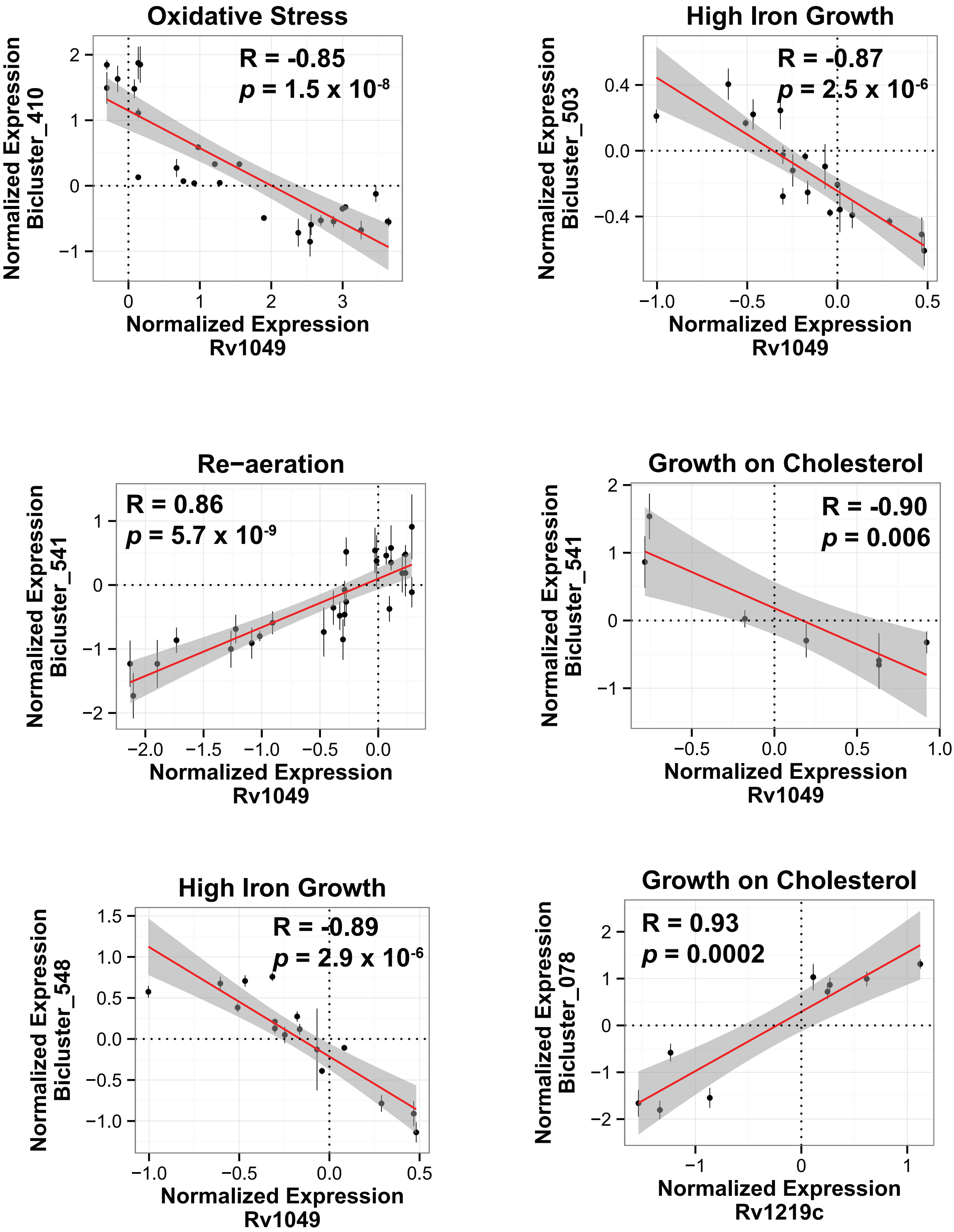

Supplemental data file S6.

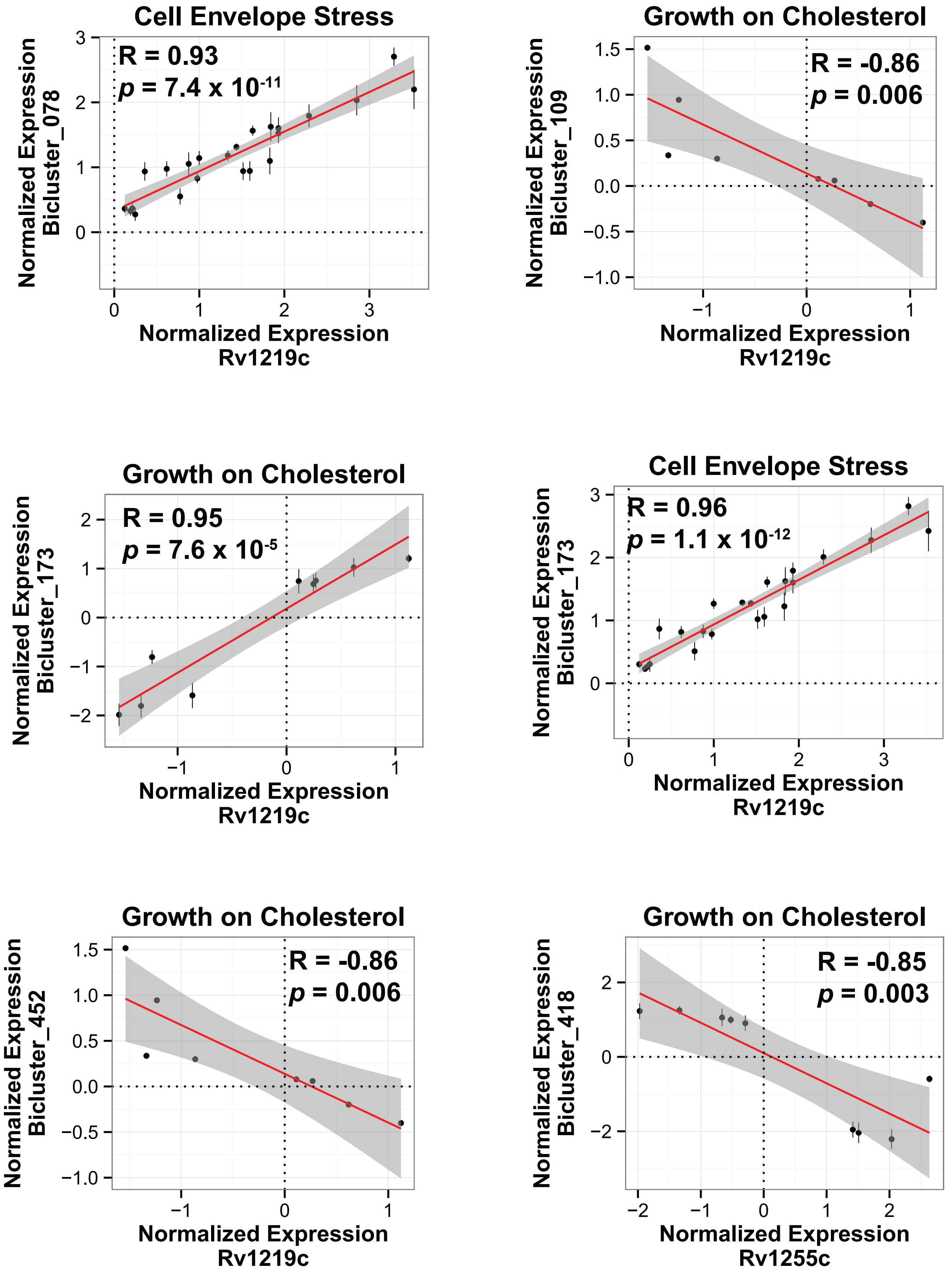

Supplemental data file S6.

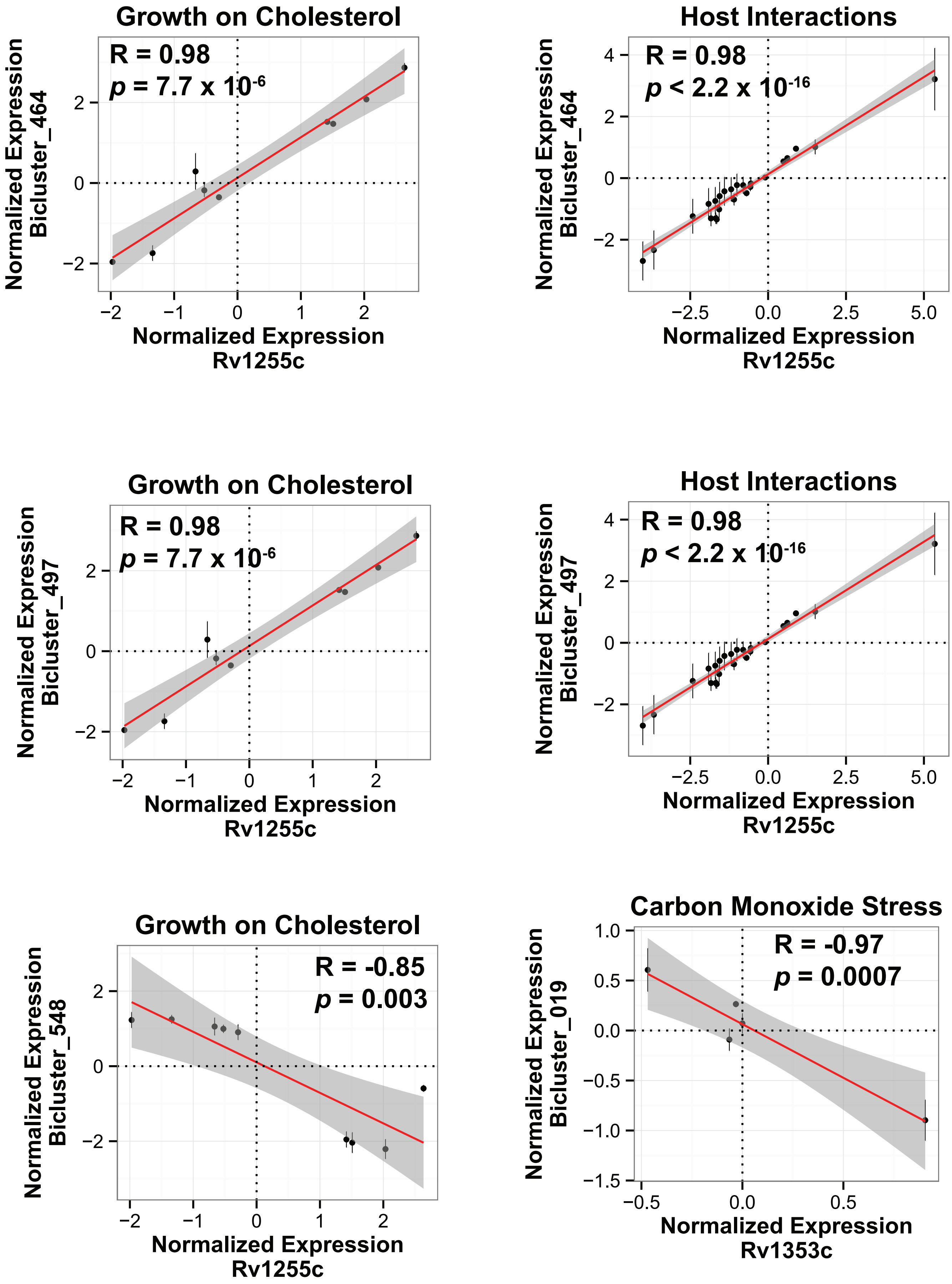

Supplemental data file S6.

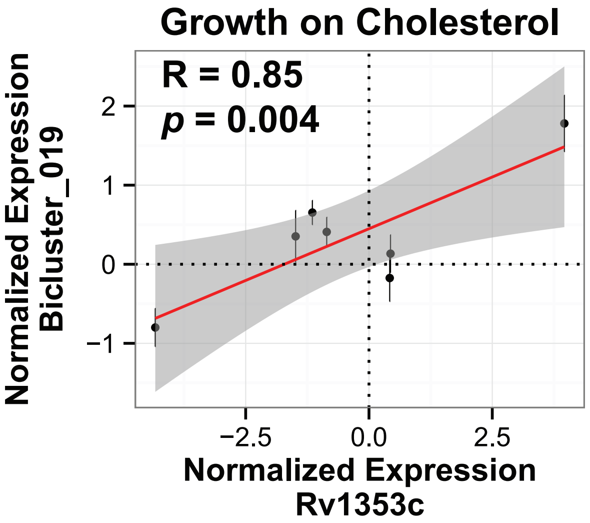

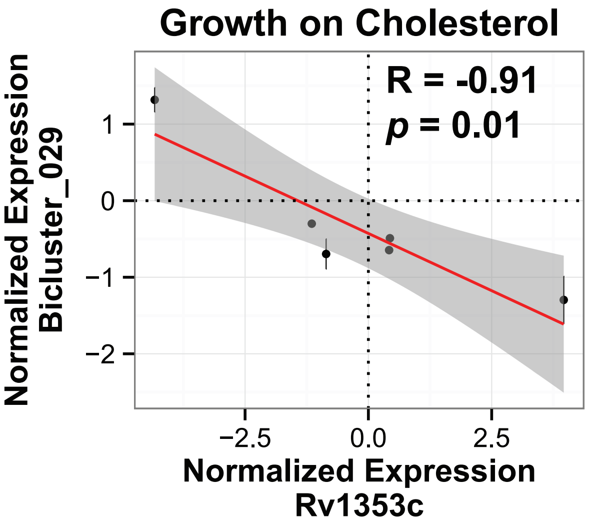

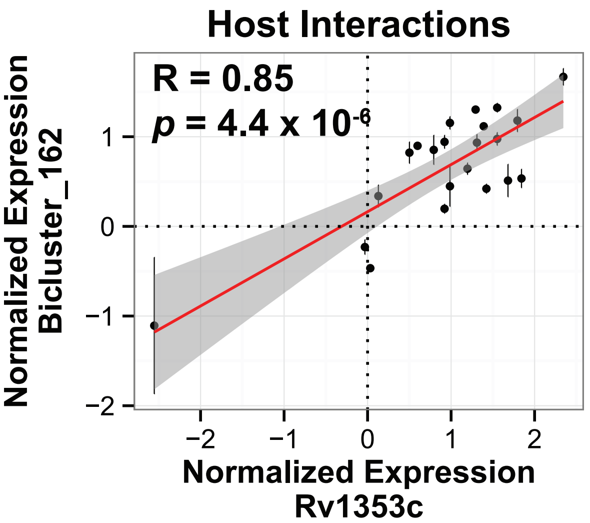

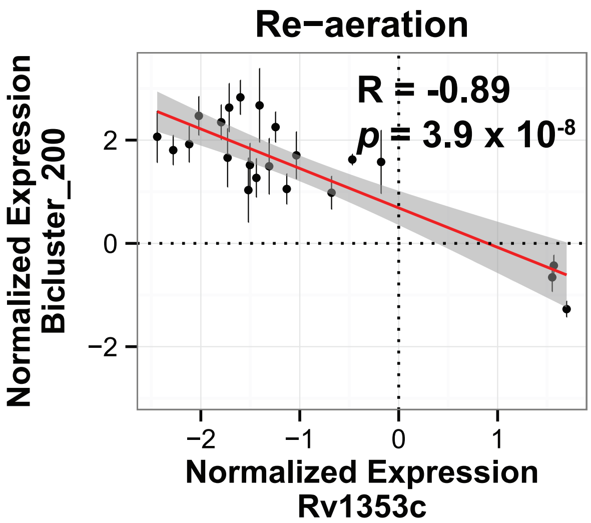

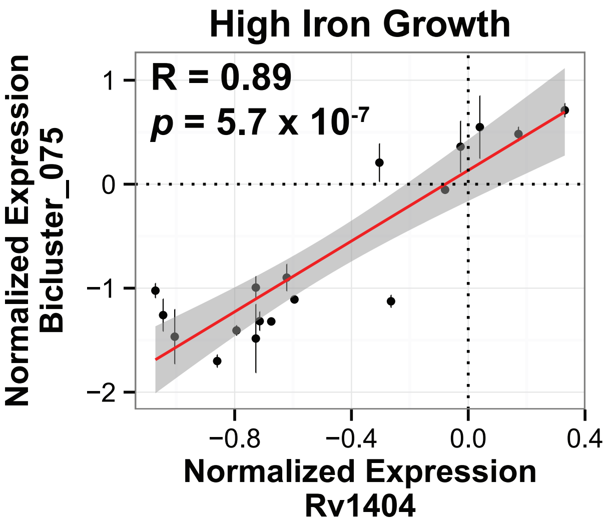

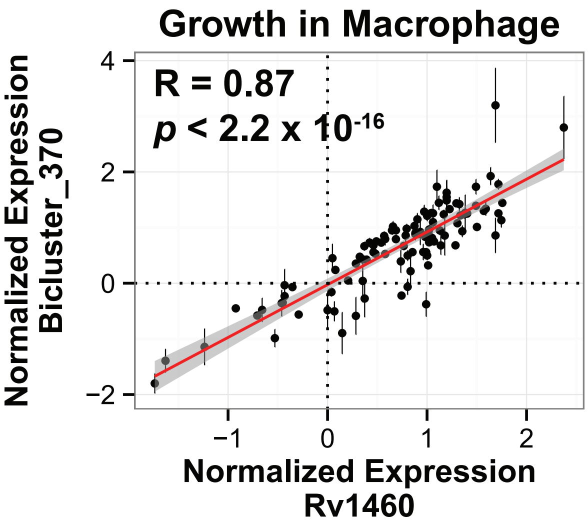

Supplemental data file S6.

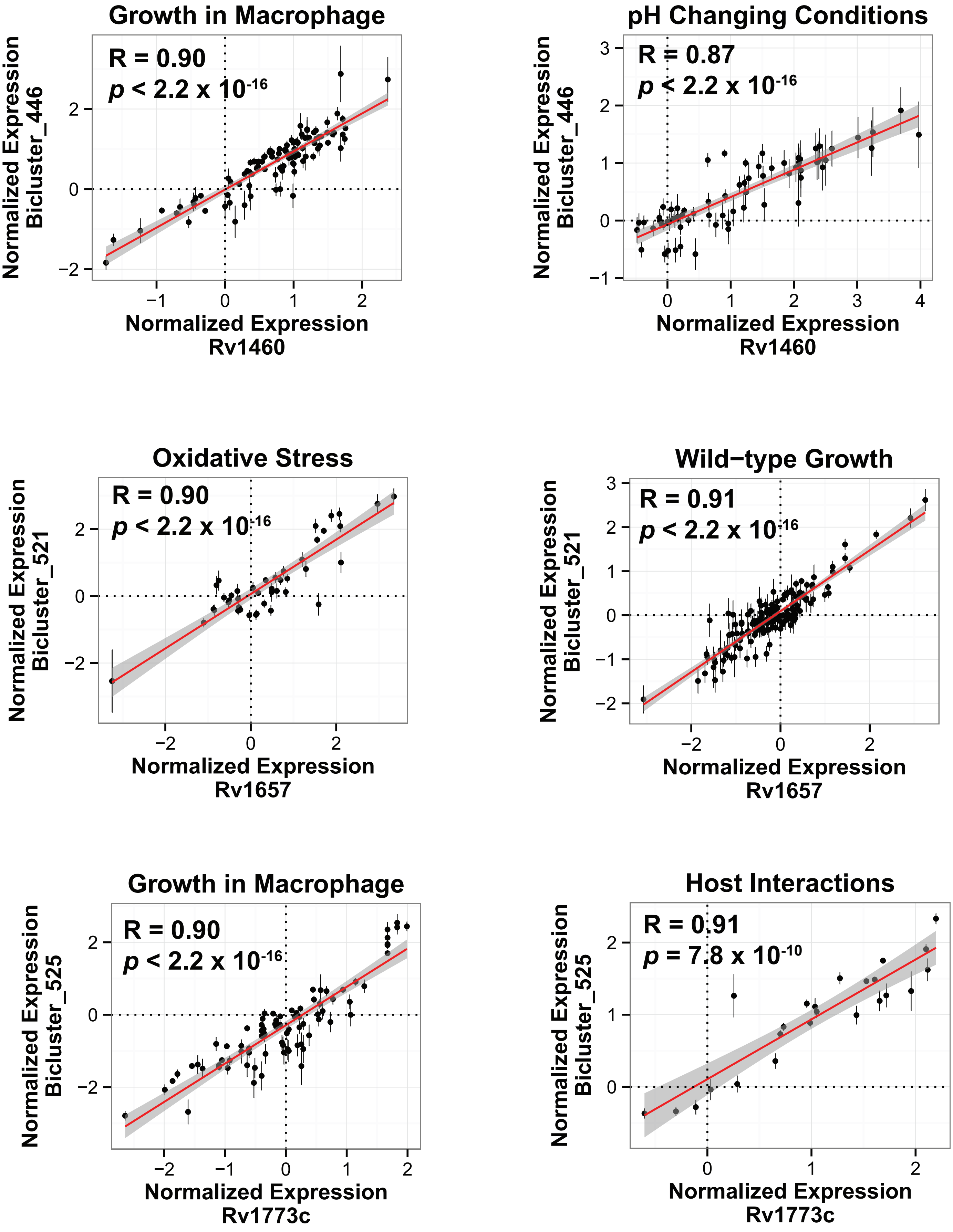

Supplemental data file S6.

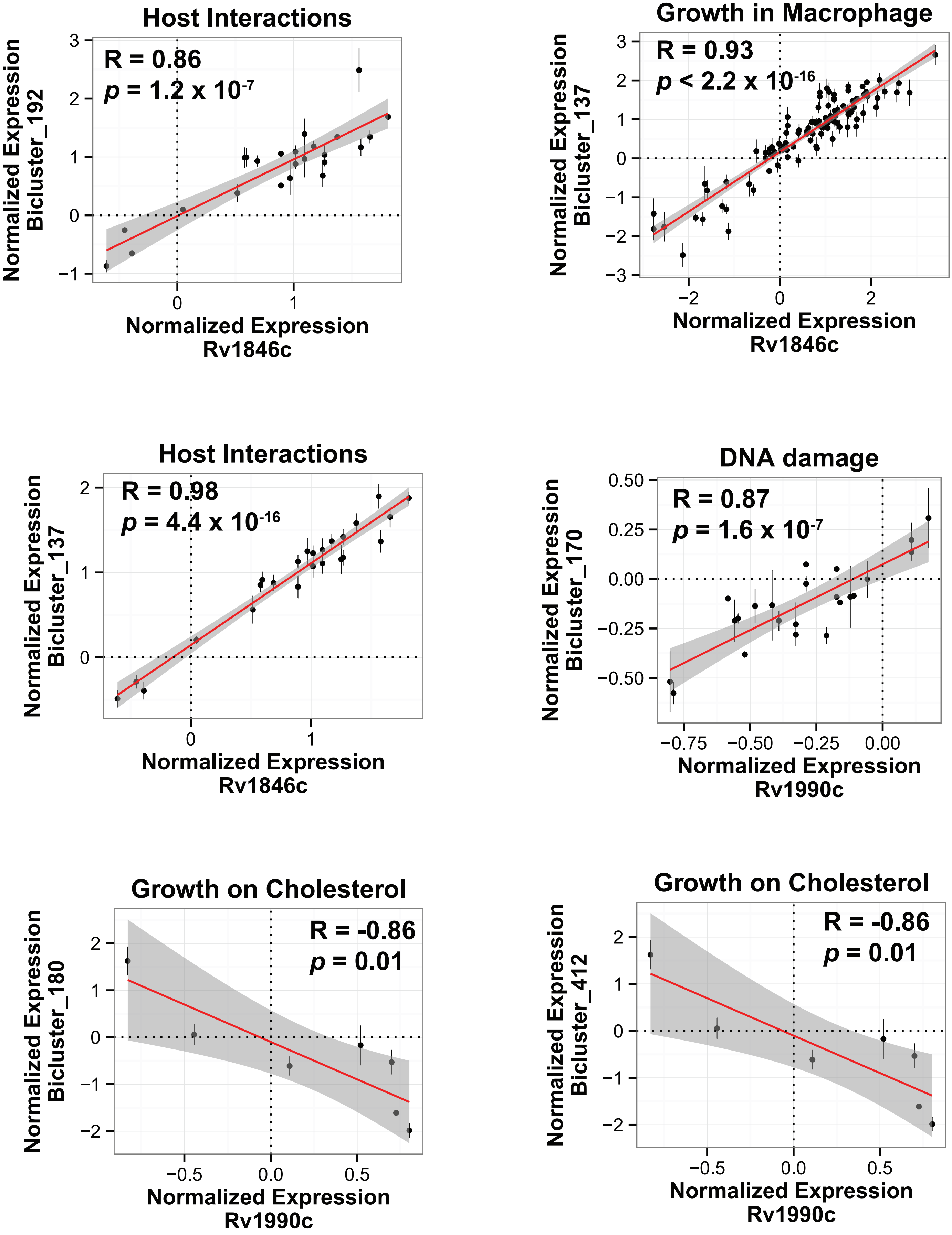

Supplemental data file S6.

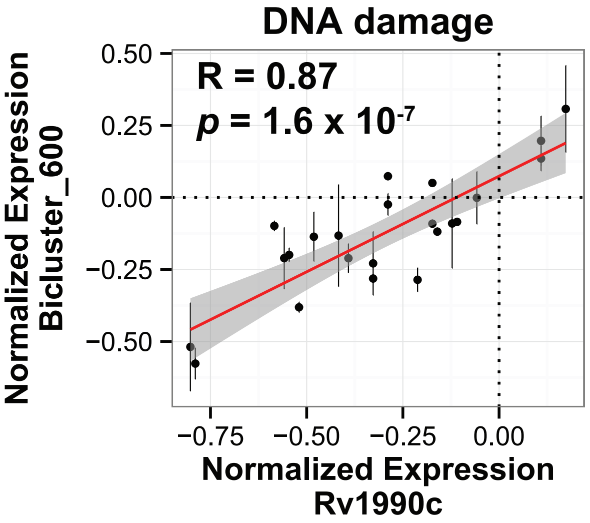

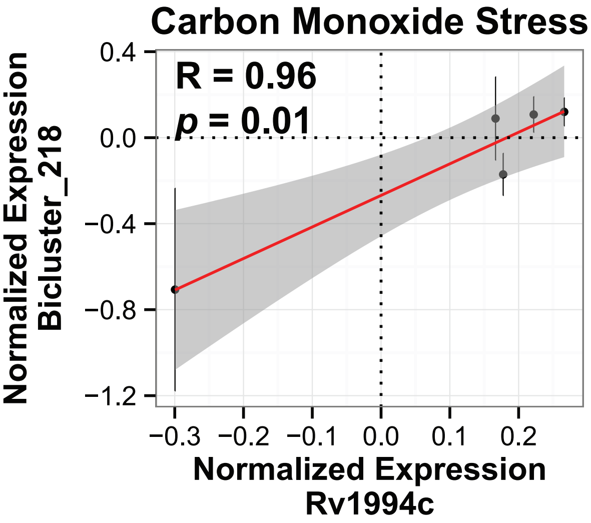

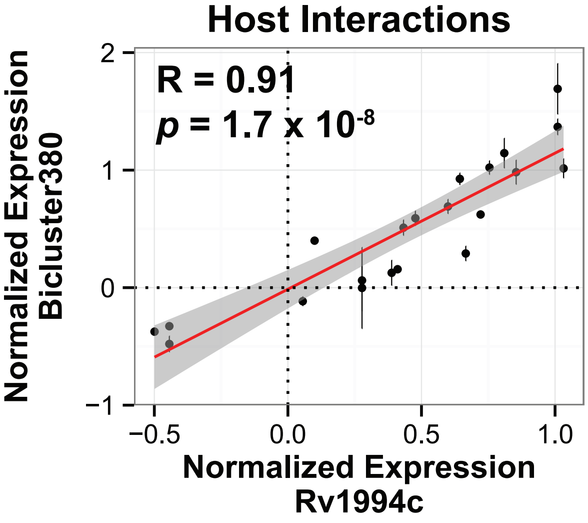

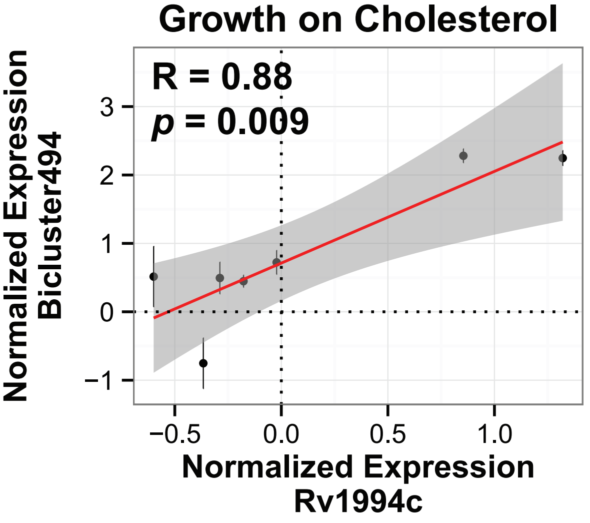

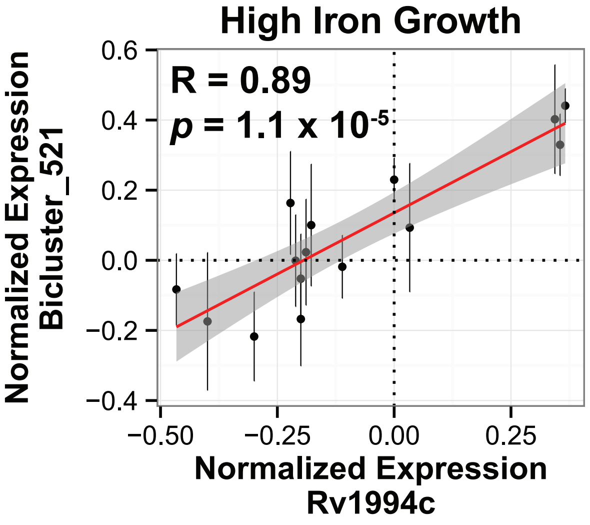

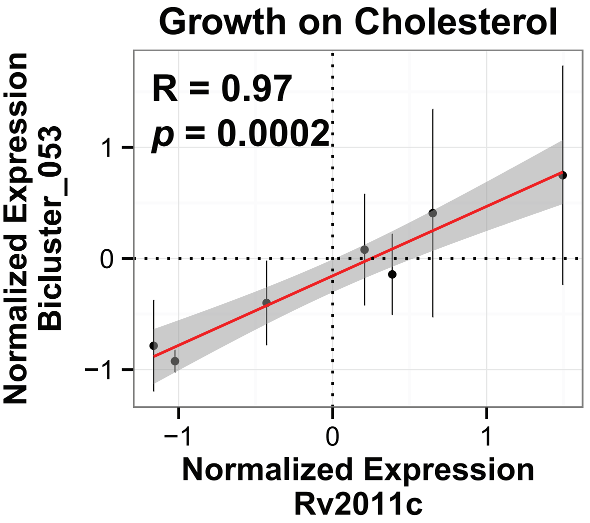

Supplemental data file S6.

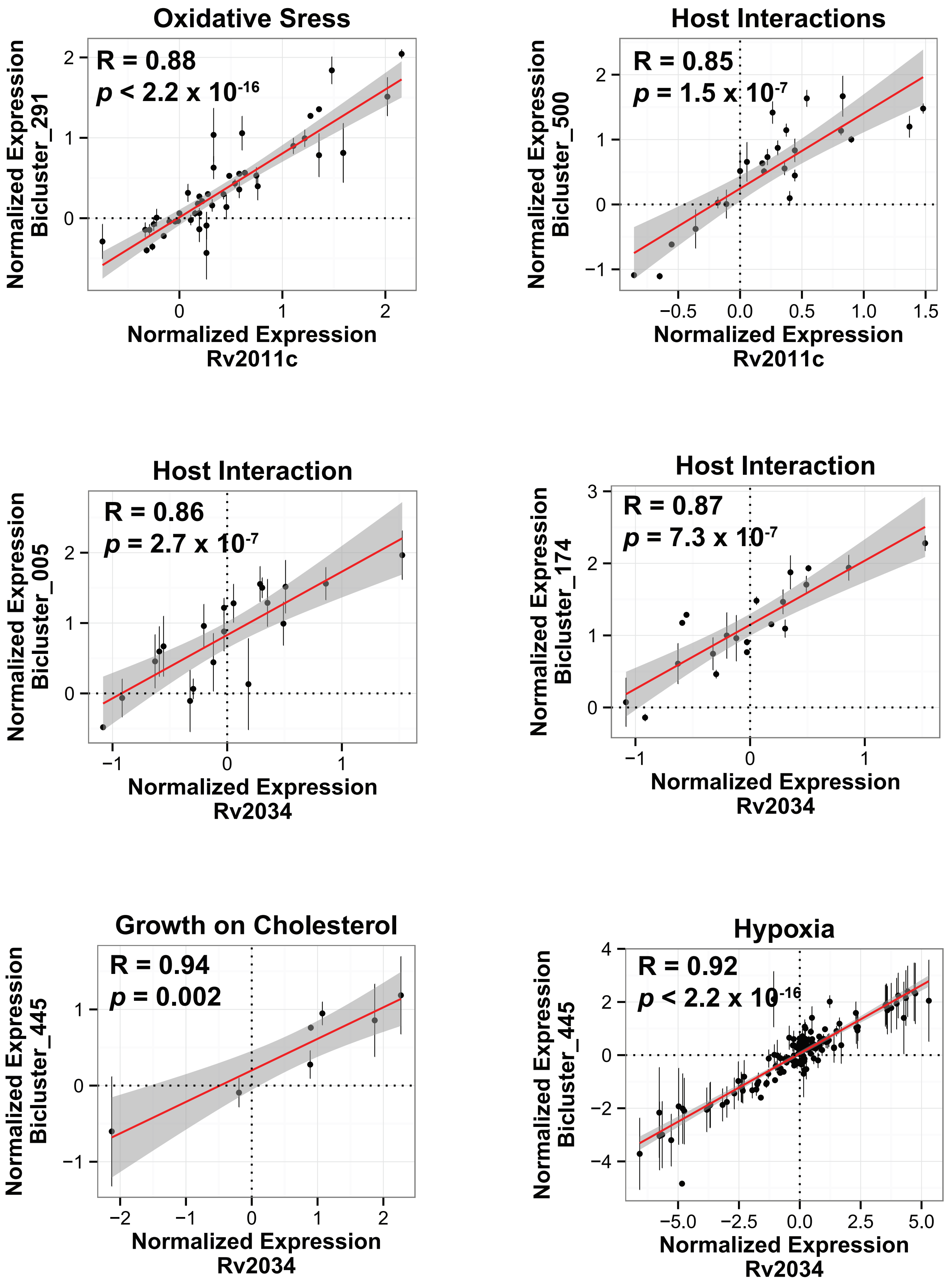

Supplemental data file S6.

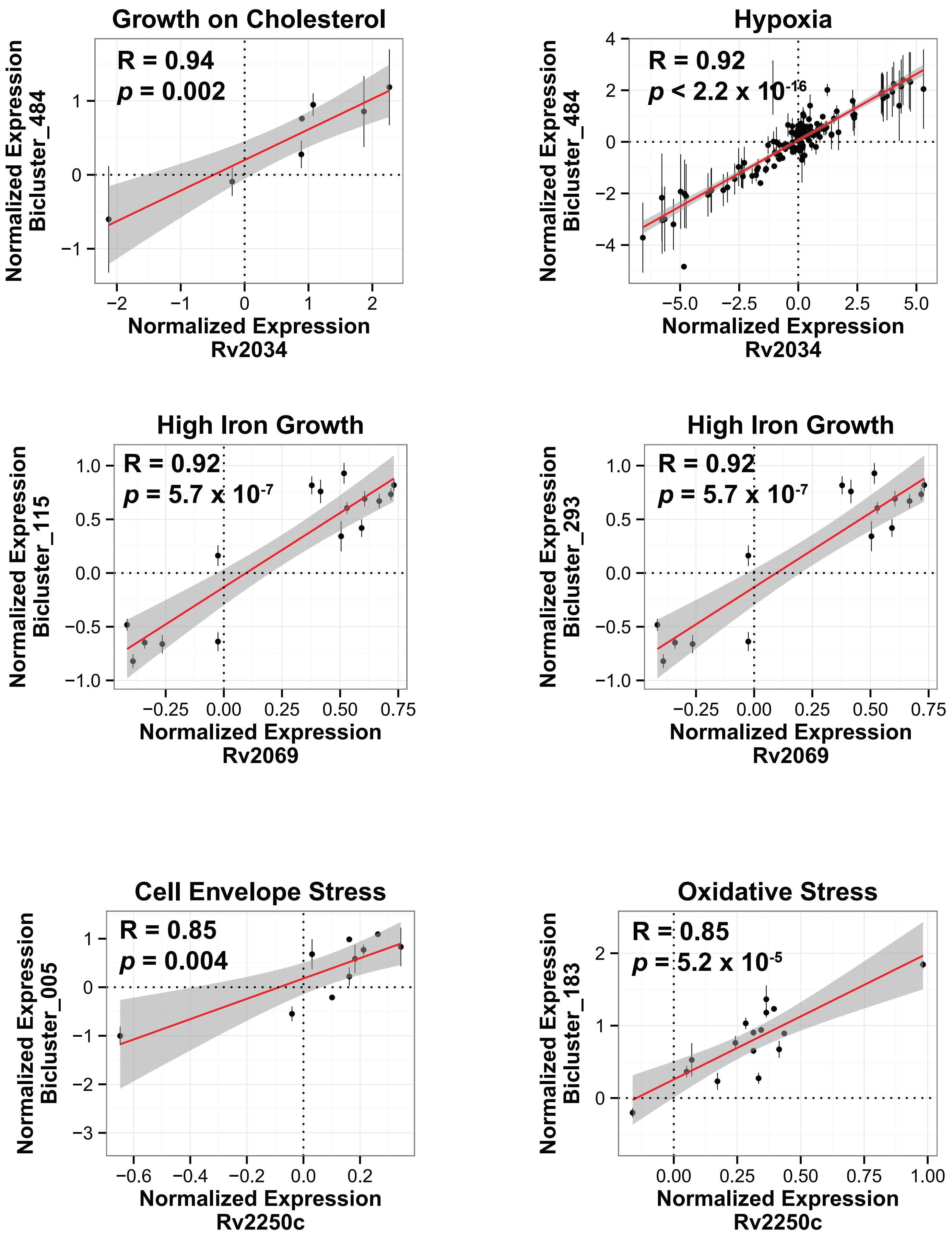

Supplemental data file S6.

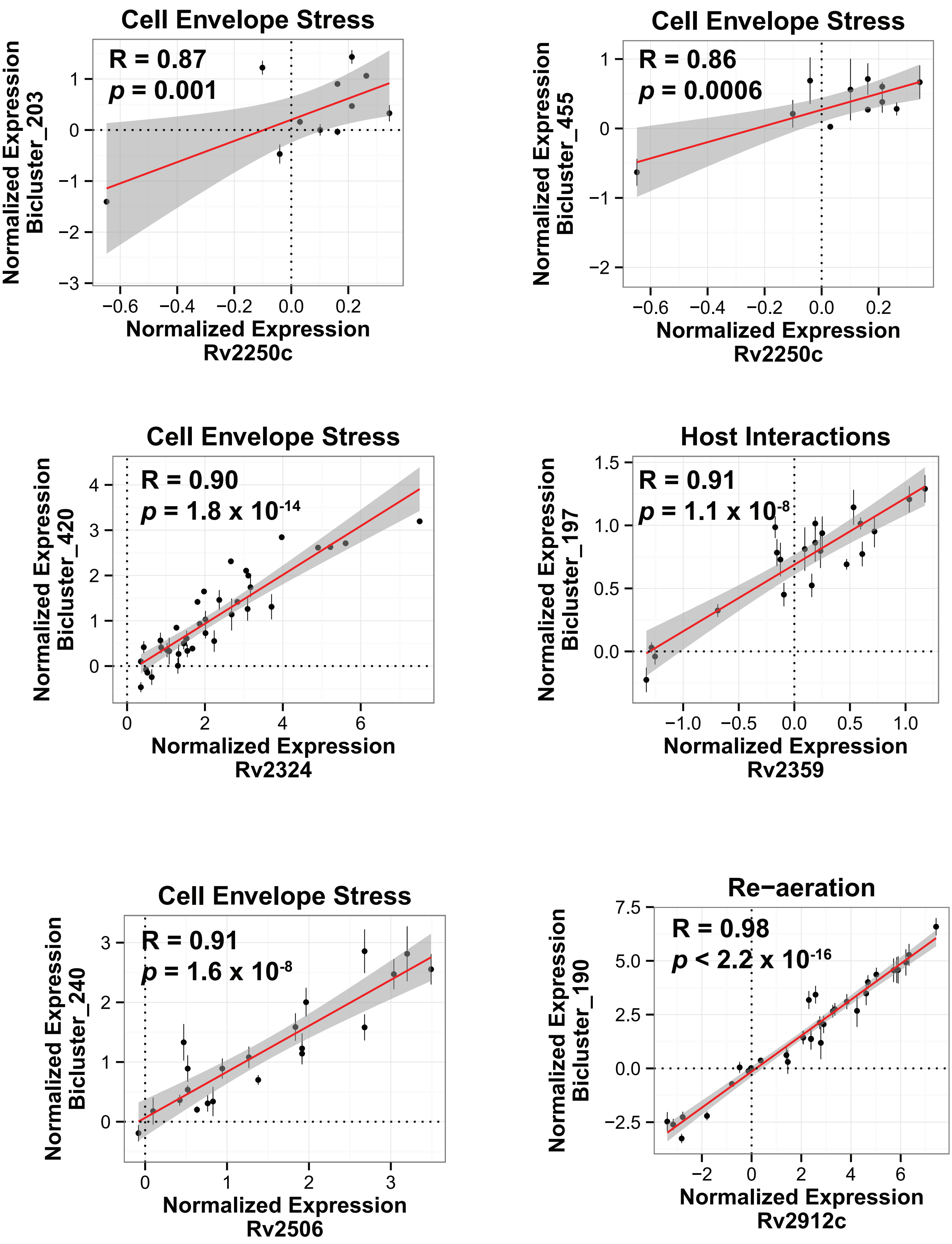

Supplemental data file S6.

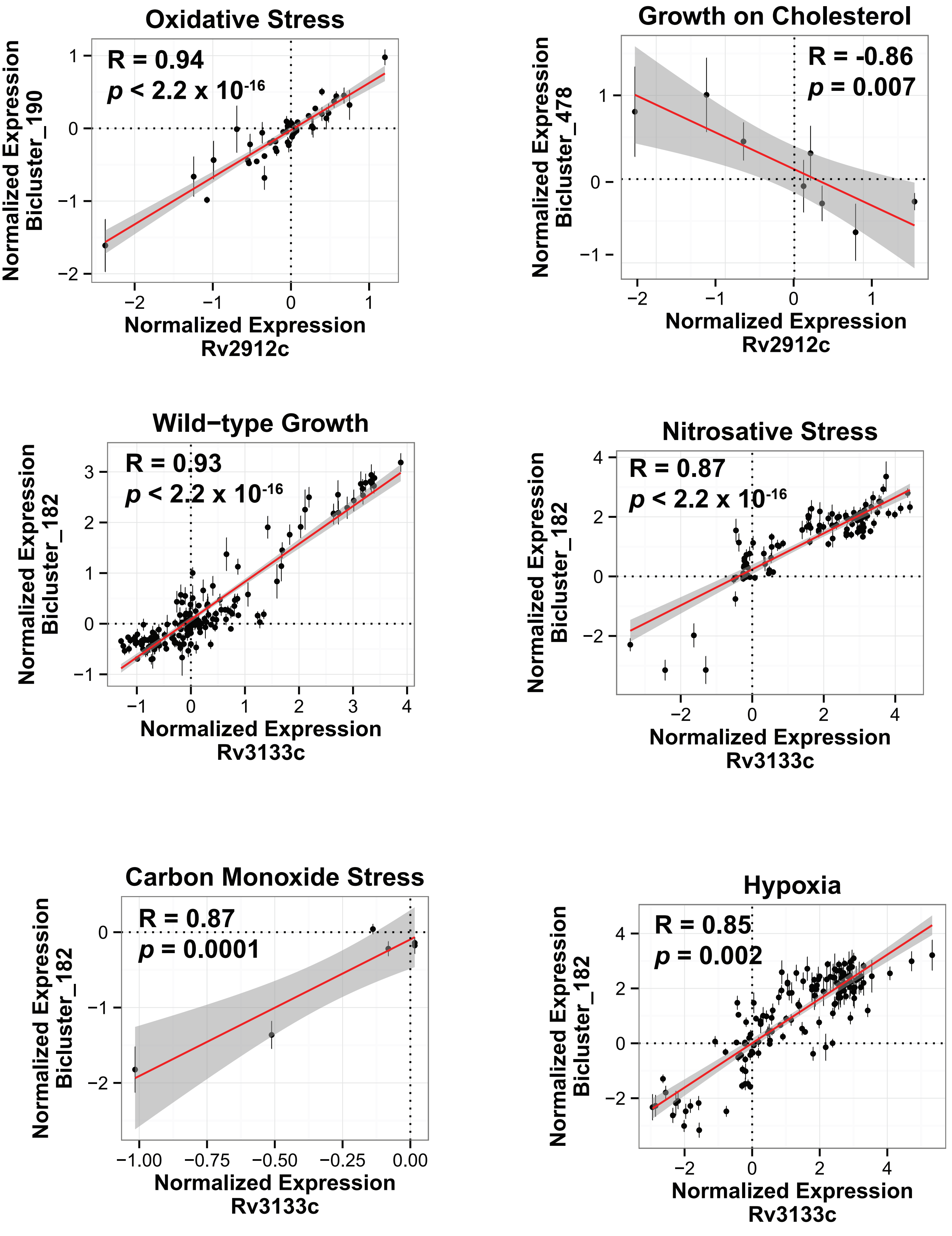

Supplemental data file S6.

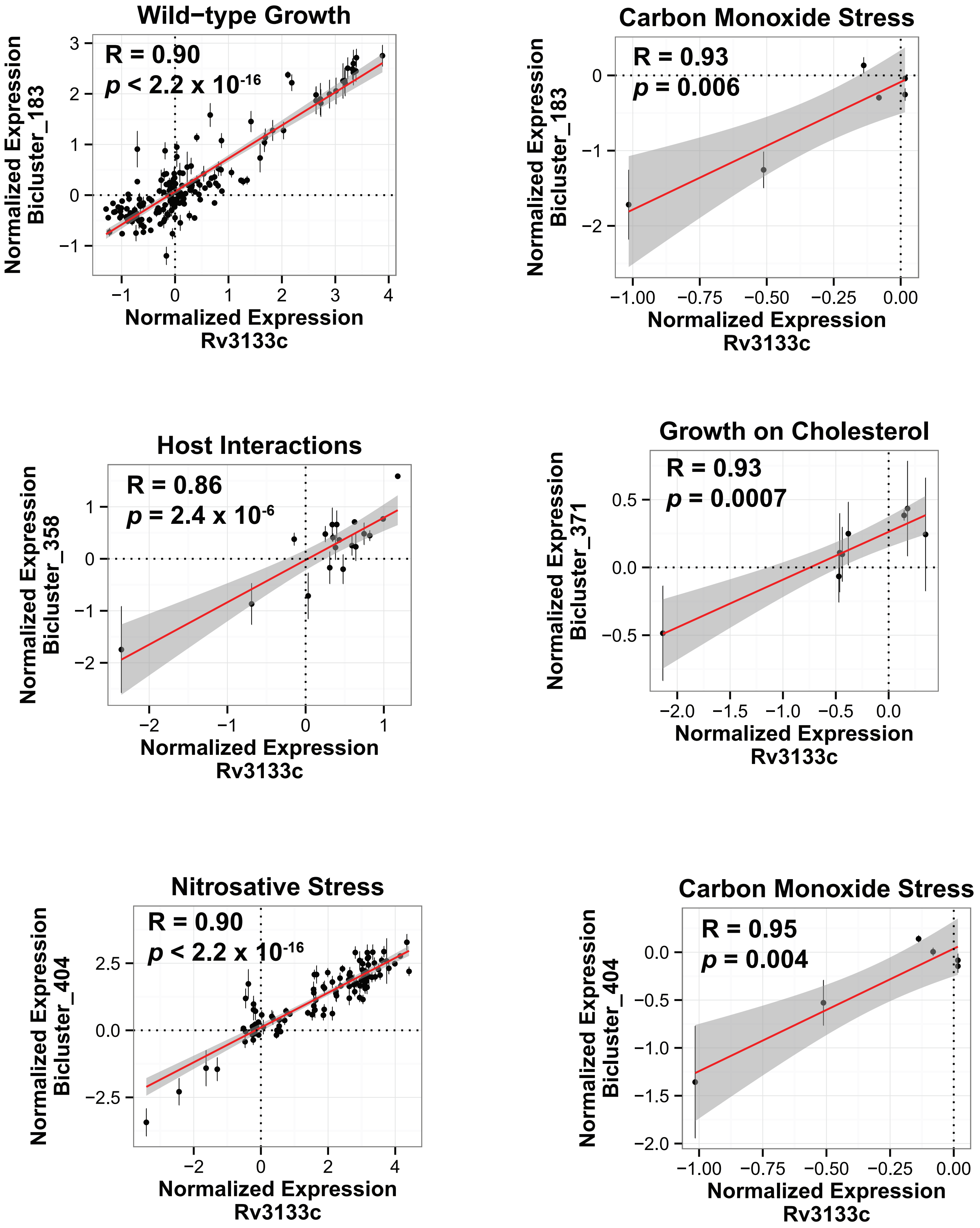

Supplemental data file S6.

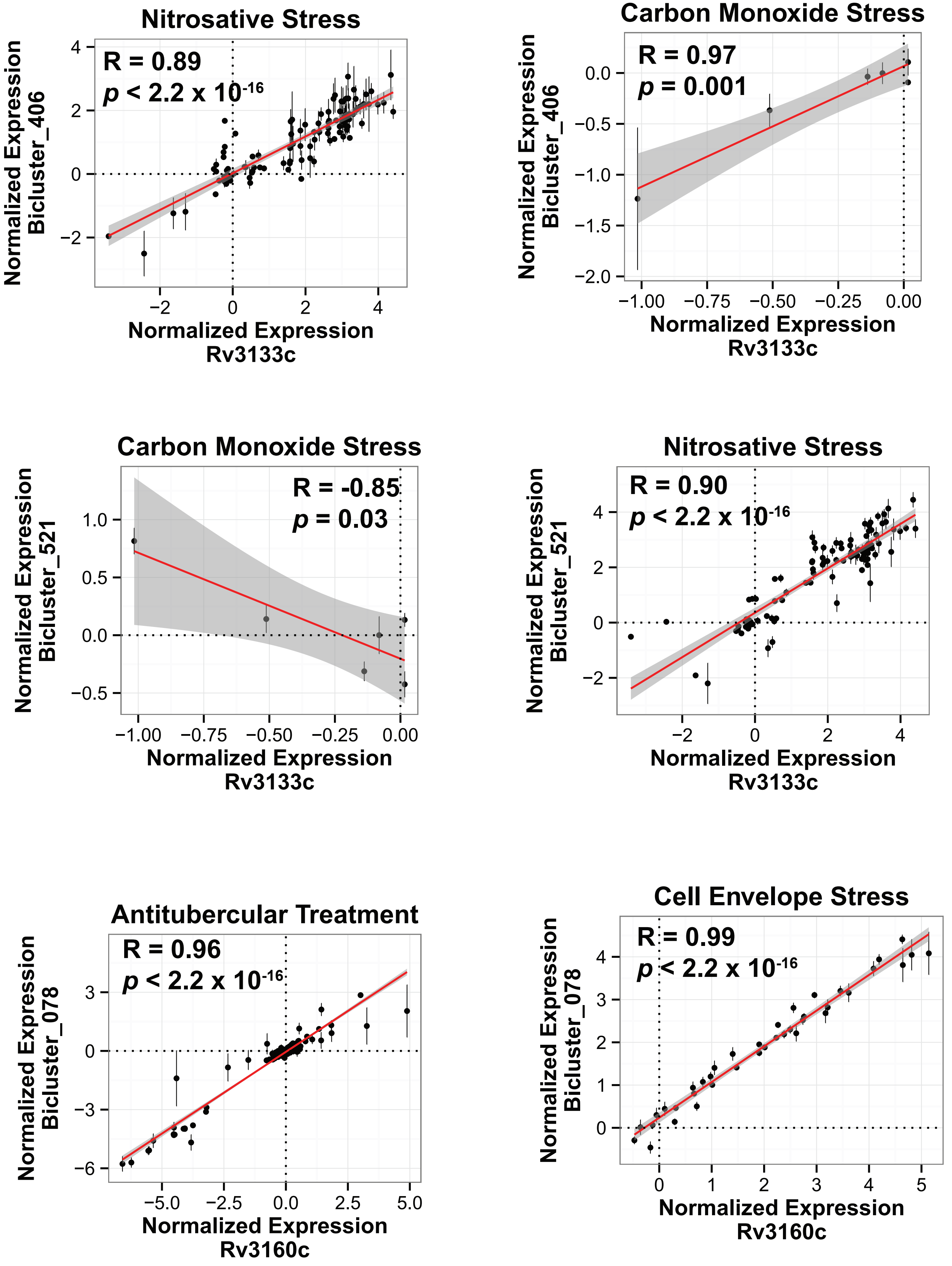

Supplemental data file S6.

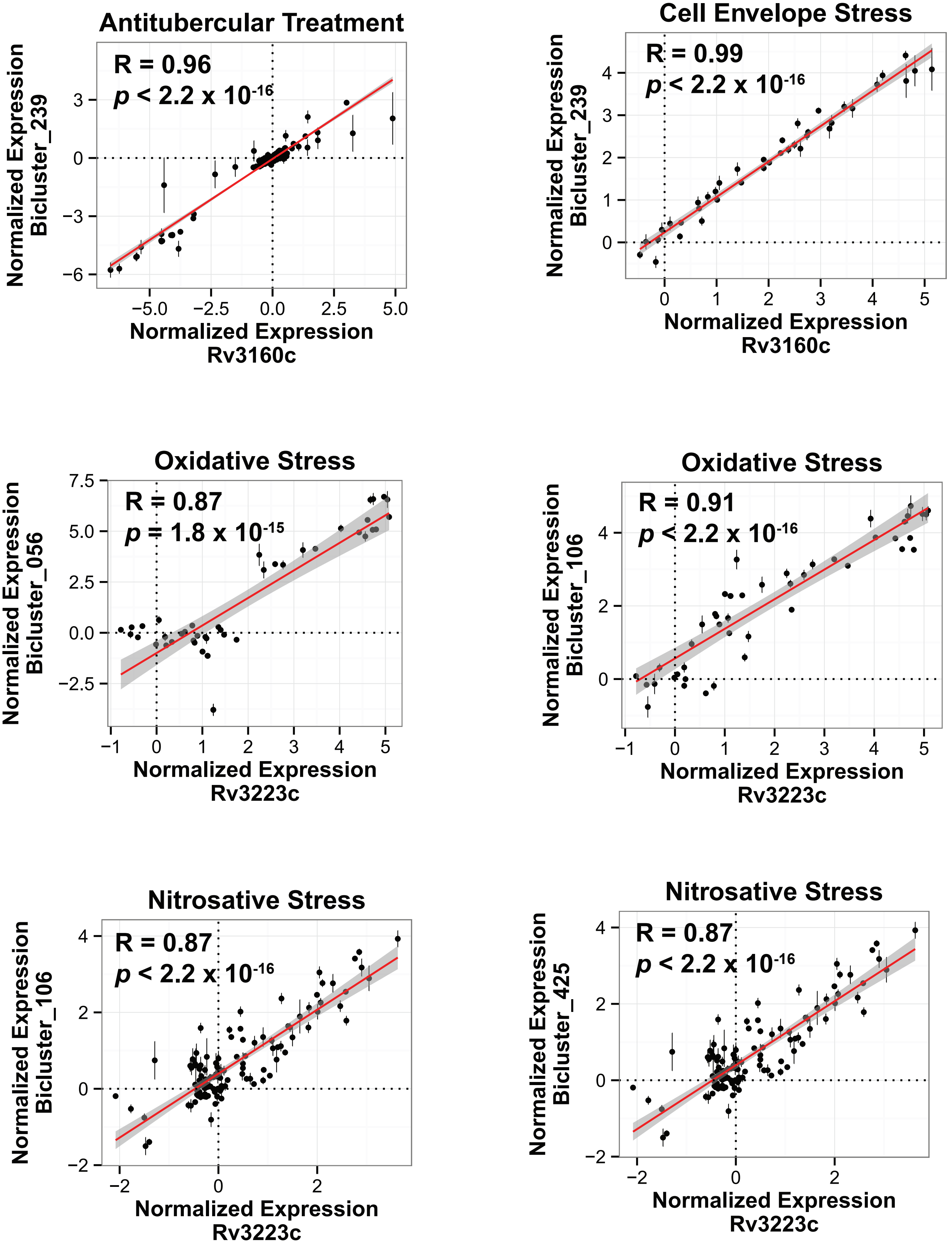

Supplemental data file S6.

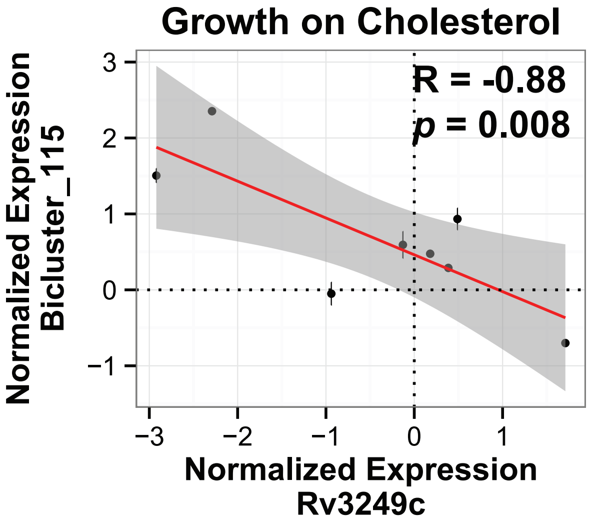

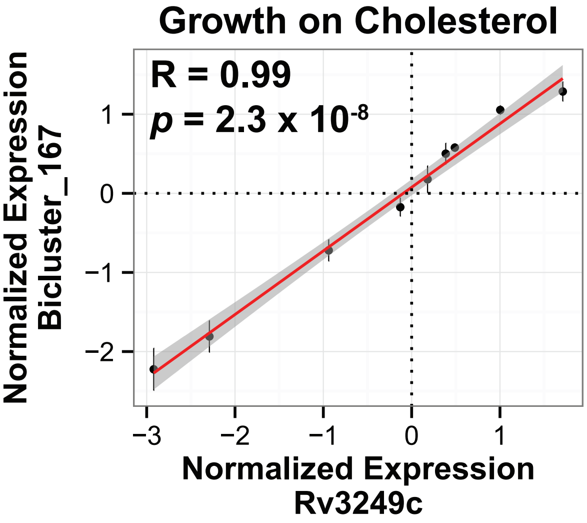

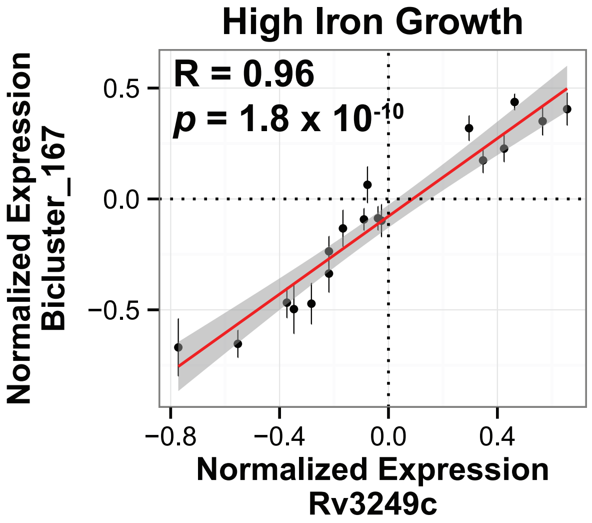

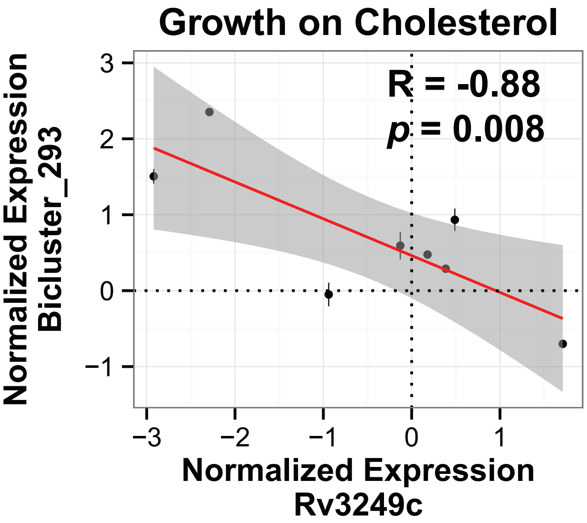

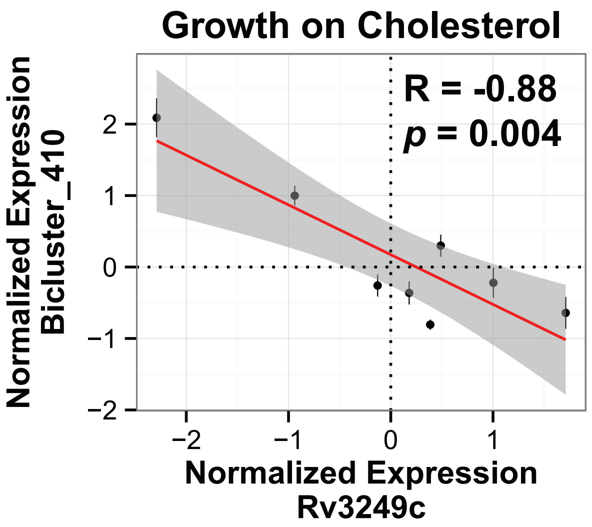

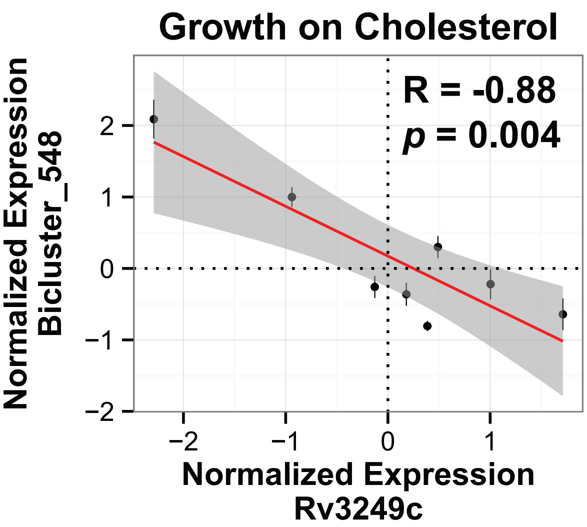

Supplemental data file S6.

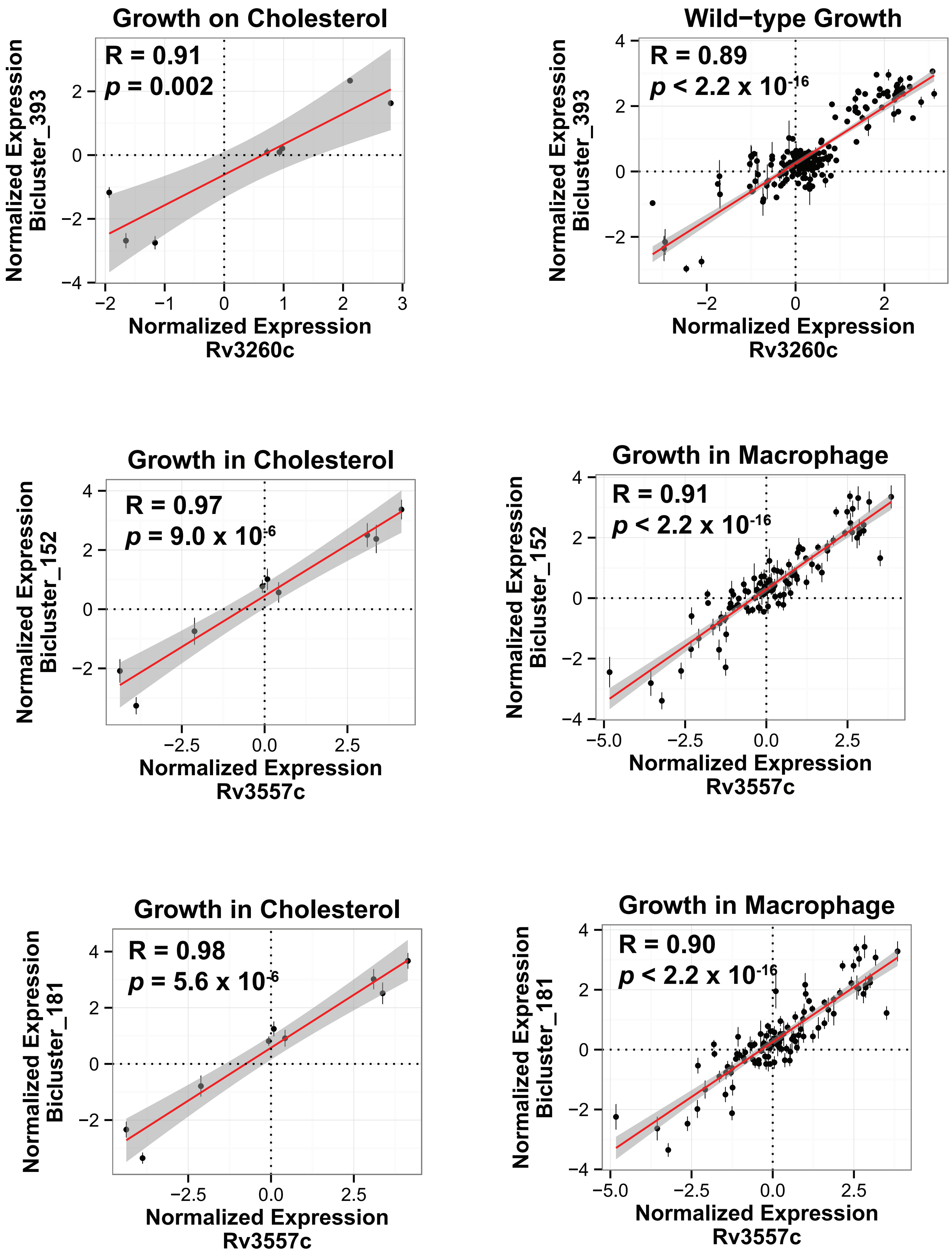

Supplemental data file S6.

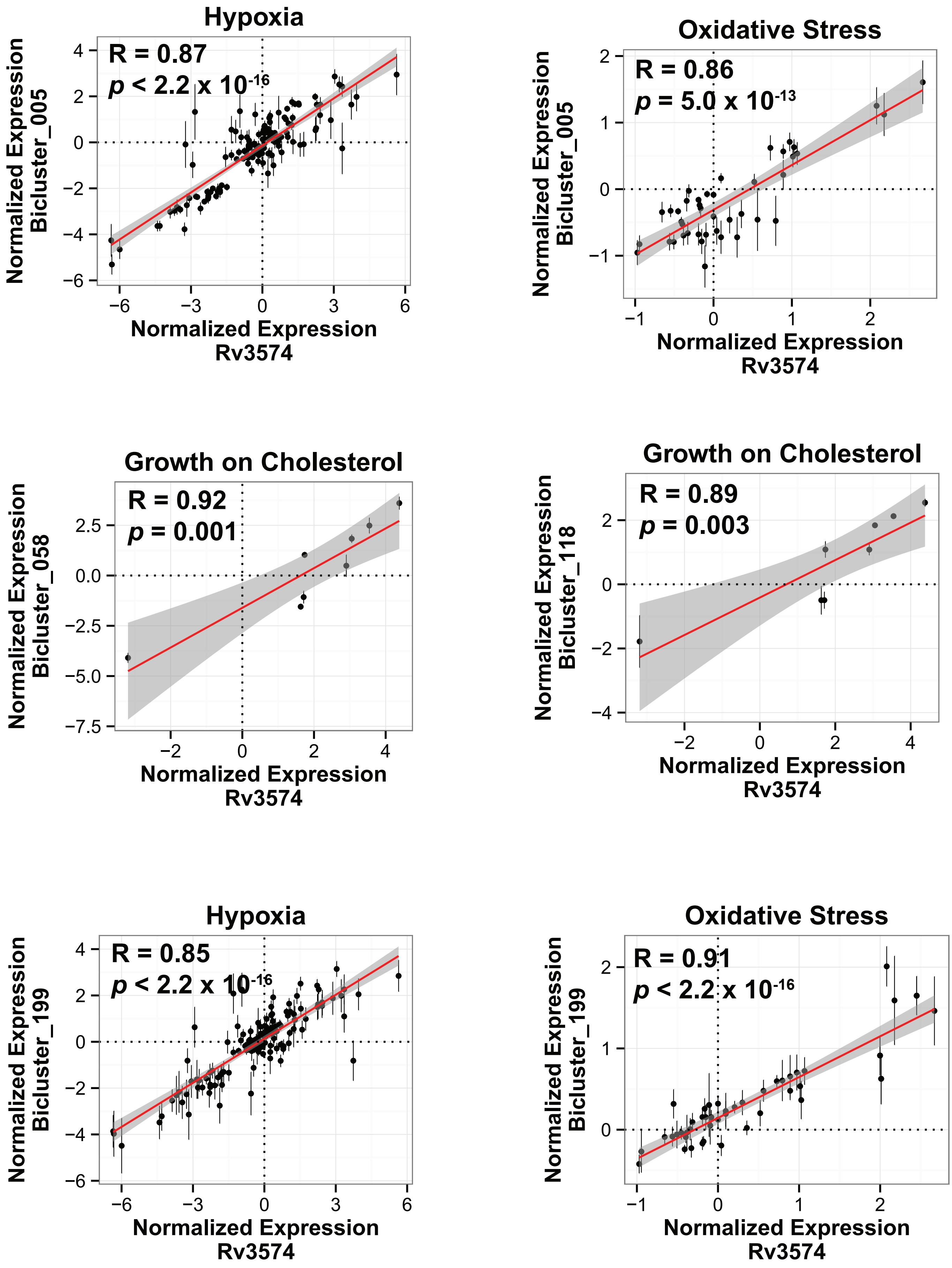

Supplemental data file S6.

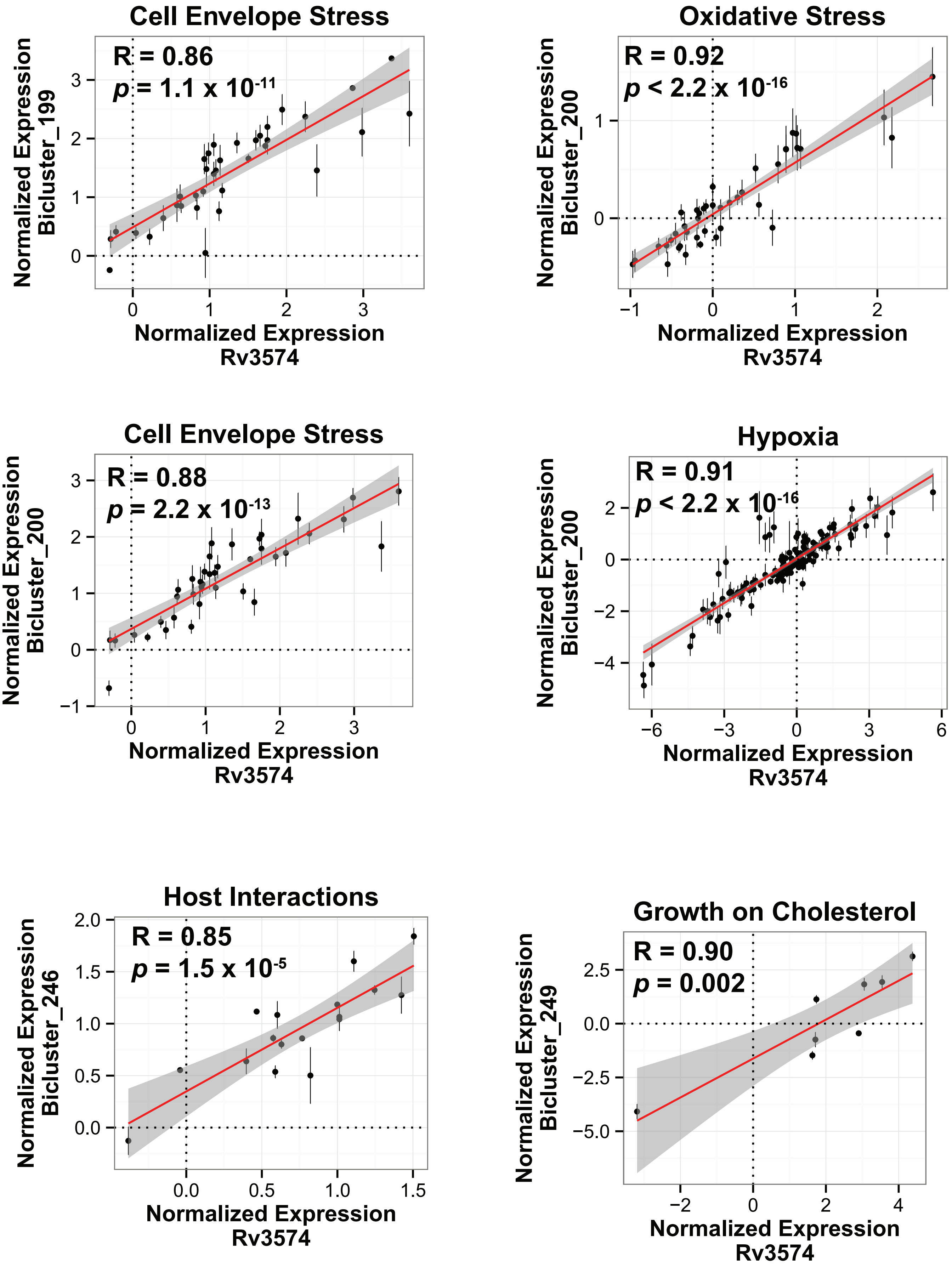

Supplemental data file S6.

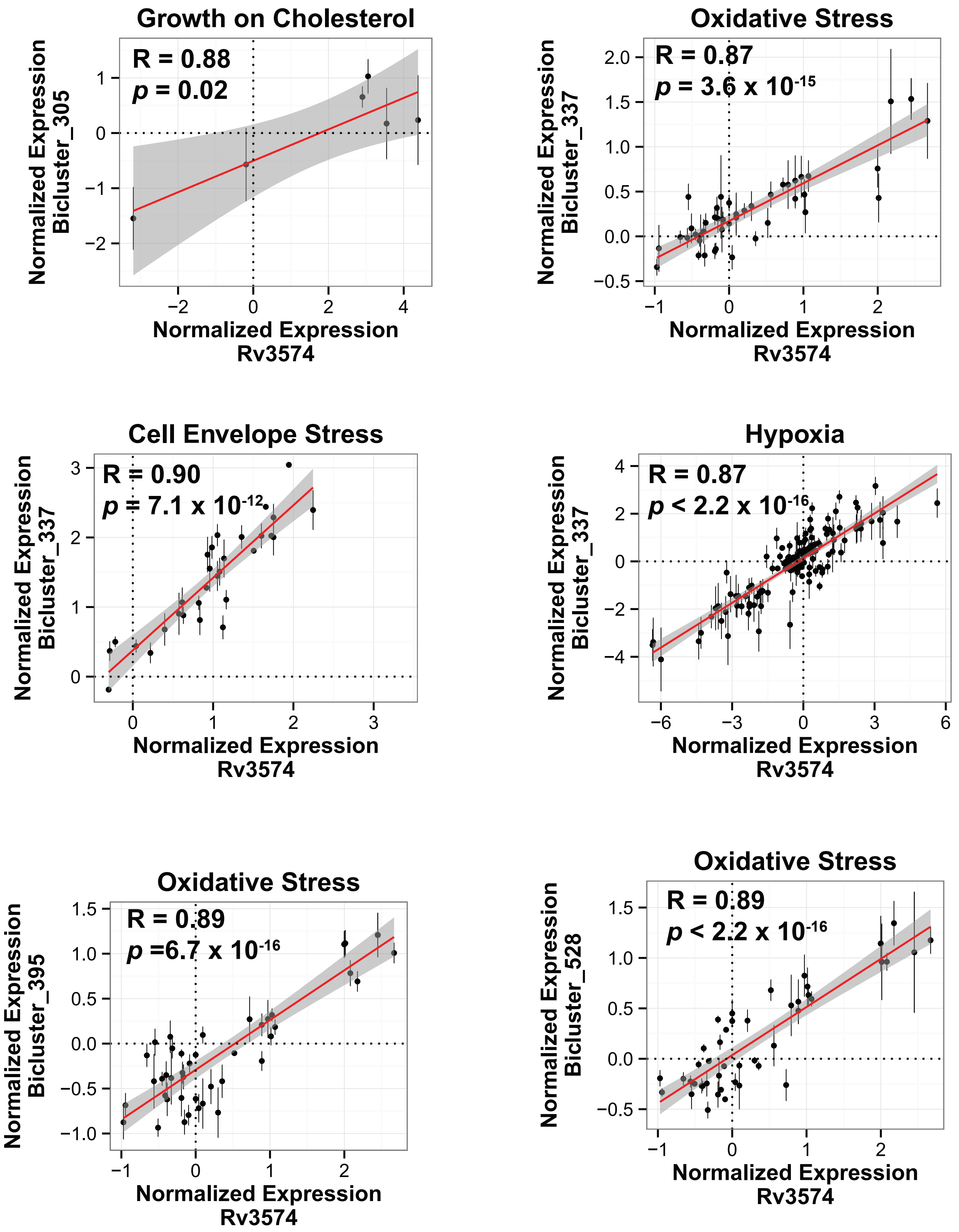

Supplemental data file S6.

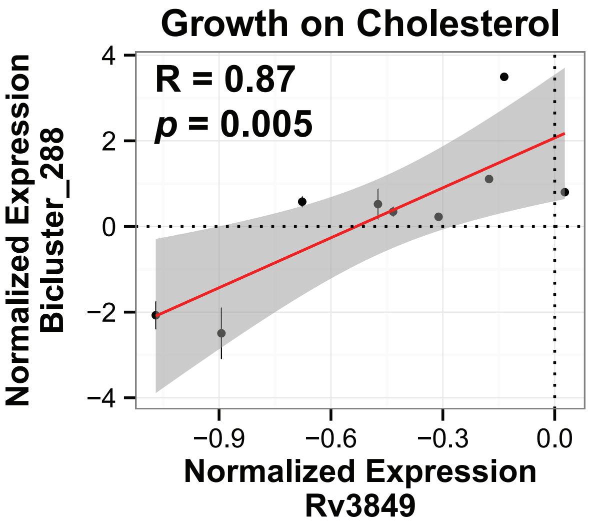

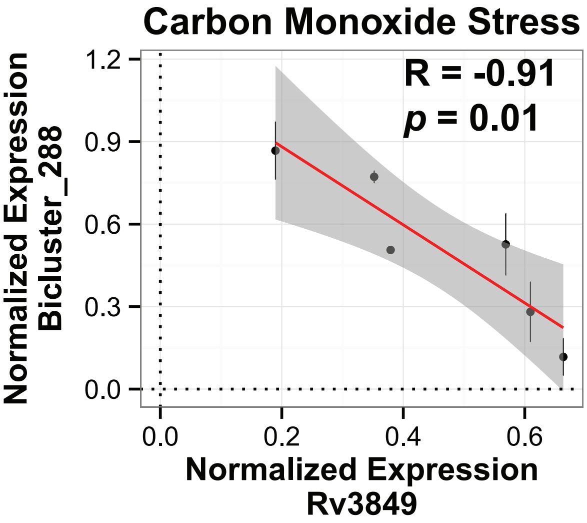

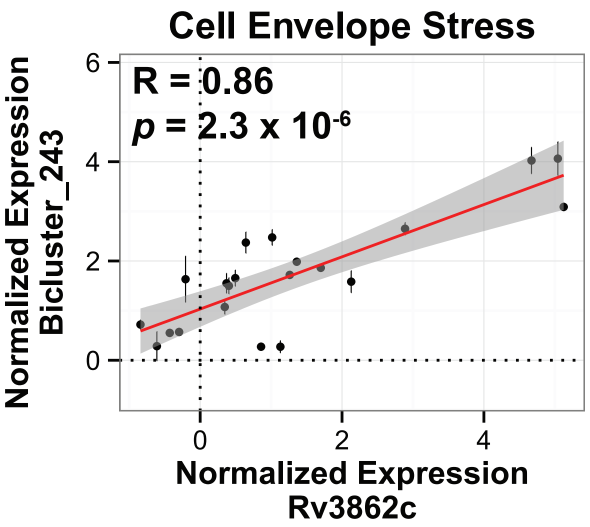

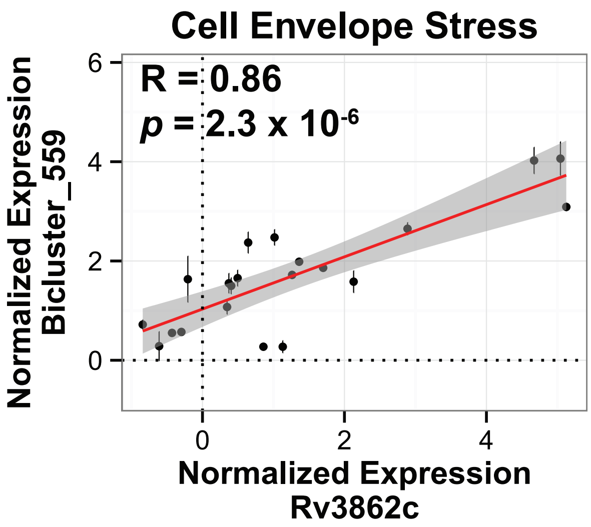

Supplemental data file S6.

Figure S2. The conditional regulation of KstR biclusters. The scatter plots show the correlation of expression for KstR versus the median correlation of gene members of bicluster_337, bicluster_200, and bicluster_199 under cell envelope stress, hypoxia, and oxidative stress conditions. The pearson's correlation coefficient (R) and *p*-value (*p*) are indicated for each plot. Error bars show the standard deviation of bicluster gene expression.

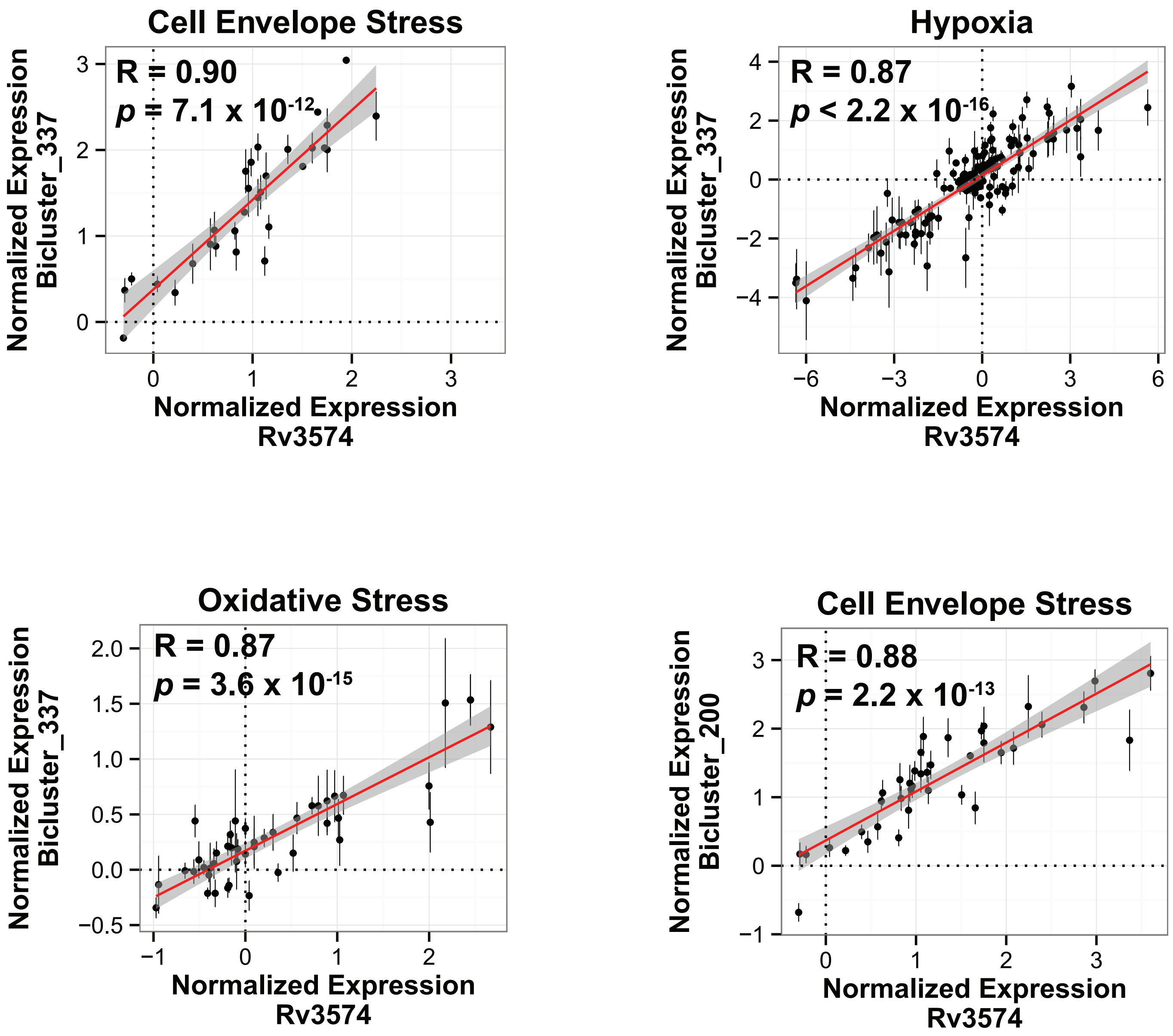

Supplemental data file S6.

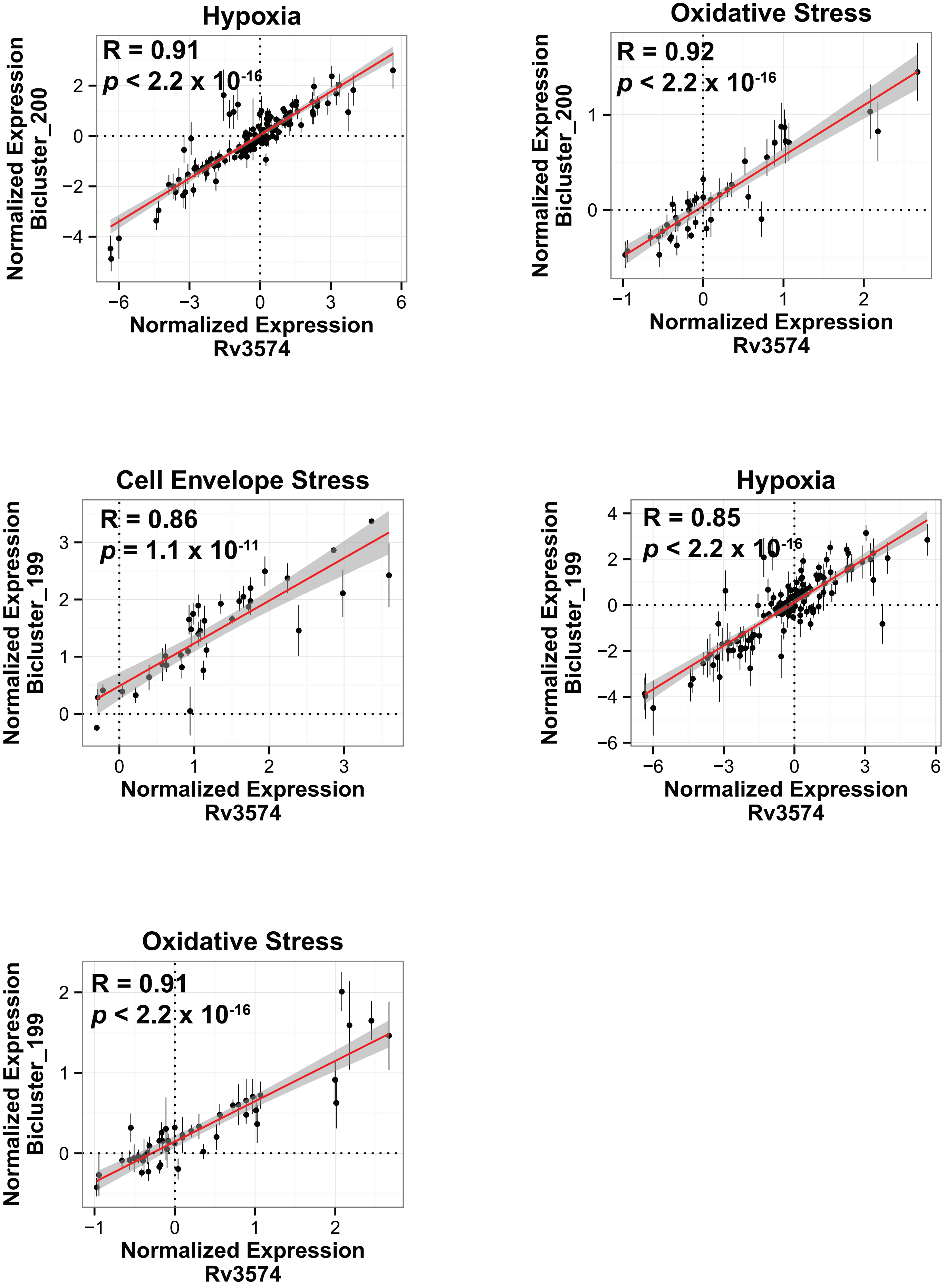

Supplement: SUPPLEMENTARY DATA [file supp_gku777_nar-01898-h-2014-File008.zip › PetersonEJR.Supp.data.file.S6.pdf]
