# Supplementary material for: A high-resolution network model for global gene regulation in Mycobacterium tuberculosis
Source: Nucleic Acids Res. 2014 Sep 17;42(18):11291–303. doi: 10.1093/nar/gku777 (PMC4191388; doi:10.1093/nar/gku777)
Supplement: SUPPLEMENTARY DATA [file supp_gku777_nar-01898-h-2014-File008.zip › PetersonEJR.Supp.data.file.S4.pdf]

**Supplemental data file S4**. Significant similarity between motifs detected by cMonkey and those identified through ChIP-Seq analysis.

| | Bicluster, Motif | Bicluster, E.value | Bicluster Logo | Match.FDR q.value | TF, Motif | TF E.value | TF Logo |
|---|---|---|---|---|---|---|---|
| 1 | 200, 1 | 8.3e-28 | 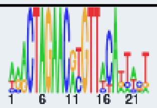 | 1.77808e-06 | Rv3574, 1 | 0.003 | 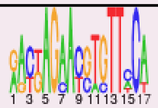 |
| 2 | 118, 1 | 2.2e-08 | 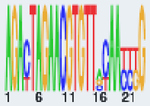 | 1.77808e-06 | Rv3574, 1 | 0.003 | 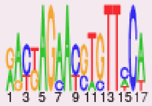 |
| 3 | 267, 1 | 1.1e-17 | 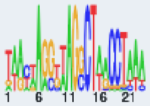 | 3.77685e-06 | Rv0576, 1 | 0.13 | 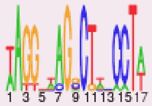 |
| 4 | 528, 1 | 0.00023 | 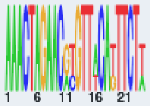 | 6.69509e-06 | Rv3574, 1 | 0.003 | 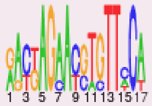 |
| 5 | 77, 1 | 37 | 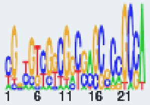 | 6.51482e-05 | Rv0967, 1 | 5.6e-57 | 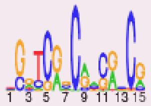 |
| 6 | 530, 1 | 8e-08 | 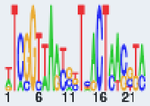 | 0.000160389 | Rv2506, 1 | 7.7e-26 | 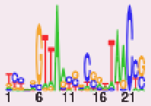 |
| 7 | 154, 1 | 6.5 | 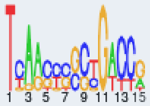 | 0.000203476 | Rv1219c, 1 | 1.4e-89 | 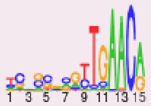 |
| 8 | 182, 1 | 2.1e-23 | 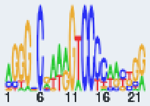 | 0.000614901 | Rv3133c, 1 | 1.1e-107 | 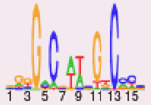 |
| 9 | 166, 2 | 0.004 | 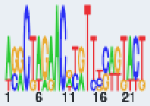 | 0.00118546 | Rv3574, 1 | 4.5e-49 | 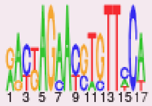 |
| 10 | 305, 1 | 6.7e-11 | 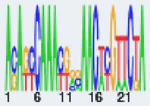 | 0.0015478 | Rv3574, 1 | 4.3e-296 | 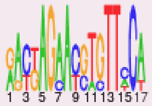 |
| 11 | 395, 1 | 0.0038 | 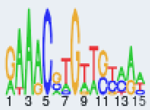 | 0.00157446 | Rv3574, 1 | 4.5e-49 | 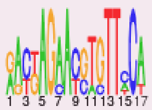 |
| 12 | 502, 1 | 2e-16 | 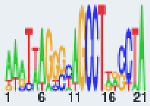 | 0.00160489 | Rv0576, 1 | 0.13 | 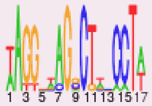 |

**Supplemental data file S4**. Significant similarity between motifs detected by cMonkey and those identified through ChIP-Seq analysis (contd).

| | Bicluster, Motif | Bicluster, E.value | Bicluster Logo | Match.FDR q.value | TF, Motif | TF E.value | TF Logo |
|---|---|---|---|---|---|---|---|
| 13 | 417, 1 | 3.9e-21 | 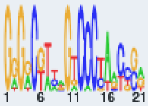 | 0.00228505 | Rv3133c, 1 | 3.1e-113 | 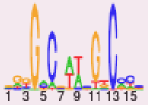 |
| 14 | 417, 2 | 0.00018 | 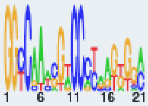 | 0.00459868 | Rv3133c, 1 | 1.1e-107 | 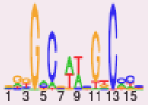 |
| 15 | 393, 2 | 14 | 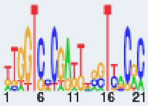 | 0.00484922 | Rv3246c, 1 | 1.3e-55 | 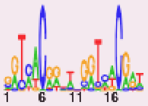 |
| 16 | 218, 2 | 2.2e-05 | 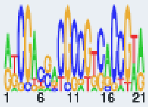 | 0.00515639 | Rv0023, 1 | 1.3e-15 | 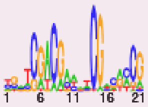 |
| 17 | 530, 1 | 8e-08 | 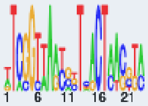 | 0.00576622 | Rv0576, 1 | 1.7e-13 | 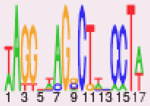 |
| 18 | 199, 1 | 1e-15 | 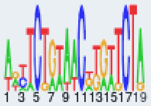 | 0.00664094 | Rv3574, 1 | 6.4e-57 | 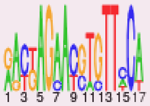 |
| 19 | 334, 1 | 0.064 | 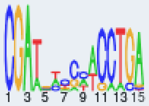 | 0.00712212 | Rv0273c, 1 | 1.2e-56 | 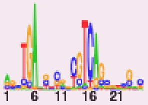 |
| 20 | 289, 1 | 4.4e-11 | 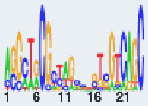 | 0.00779848 | Rv0576, 1 | 1.7e-13 | 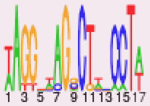 |
| 21 | 463, 2 | 3.1e-07 | 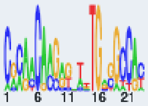 | 0.00893249 | Rv2642, 1 | 5.3e-75 | 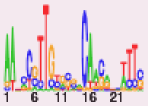 |
| 22 | 80, 1 | 2.7e-05 | 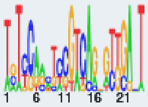 | 0.00908399 | Rv0576, 1 | 1.7e-13 | 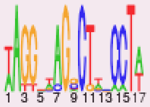 |
